# Synthesis and highly efficient light-induced rearrangements of diphenylmethylene(2-benzo[*b*]thienyl)fulgides and fulgimides

**DOI:** 10.3762/bjoc.16.149

**Published:** 2020-07-22

**Authors:** Vladimir P Rybalkin, Sofiya Yu Zmeeva, Lidiya L Popova, Valerii V Tkachev, Andrey N Utenyshev, Olga Yu Karlutova, Alexander D Dubonosov, Vladimir A Bren, Sergey M Aldoshin, Vladimir I Minkin

**Affiliations:** 1 Federal Research Centre the Southern Scientific Centre of the Russian Academy of Sciences, Rostov on Don 344006, Russian Federation; 2 Institute of Physical and Organic Chemistry, Southern Federal University, Rostov on Don 344090, Russian Federation; 3 Institute of Problems of Chemical Physics, Russian Academy of Sciences, Chernogolovka, Moscow region 142432, Russian Federation

**Keywords:** benzo[*b*]thiophene, fulgide, fulgimide, photorearrangement, X-ray diffraction

## Abstract

2-Benzo[*b*]thienyl fulgides and fulgimides containing bulky diphenylmethylene substituents were synthesized in the form of their ring-opened *E*- or *Z-*isomers. In contrast to the majority of known fulgides/fulgimides, that form colored ring-closed structures under UV irradiation, the obtained compounds undergo an irreversible transformation leading to decoloration of their solutions. This rearrangement with the formation of the dihydronaphthalene core appeared to be by 2–3 orders of magnitude more efficient than for the known diphenylmethylene(aryl(hetaryl))fulgides. The molecular structures of *E*- and *Z-*isomers and of products of the photoinduced rearrangement completed by 1,5-H shift reaction, 3a,4-dihydronaphtho[2,3-*c*]furans(pyrroles) **C**, were established based on the data of ^1^H and ^13^C NMR spectroscopy and X-ray diffraction studies.

## Introduction

Photochromic compounds are of considerable interest as molecular switches, elements of optical memory and molecular logic devices [1–4]. Fulgides and fulgimides possessing excellent thermal and photochemical stability, structurally modulated fluorescent properties and high quantum efficiencies of their light-induced pericyclic rearrangements constitute one of the most prospective and amply studied photochromic families [5–12]. A general approach to the synthesis of fulgides is based on a Stobbe condensation [13] of aromatic or heterocyclic aldehydes or ketones with methylene succinates followed by subsequent hydrolysis and dehydration processes. Under exposure of solutions or crystals of thus prepared hetaryl(aryl)-substituted dihydrofuran-2,5-diones to UV light, those bearing an isopropylidene fragment attached to the furandione moiety, usually demonstrate positive photochromism as the result of conversion to their deeply colored cyclic isomers. However, fulgides containing a diphenylmethylene group are capable of negative photoinduced rearrangements [14–17].

**Scheme 1:**
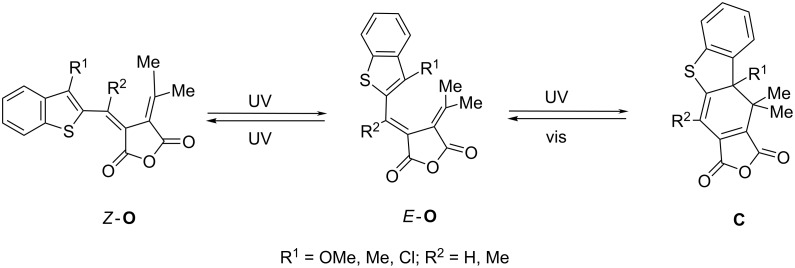
Photoisomerization of 2-benzo[*b*]thienyl fulgides.

Comparison of the photochromic properties of aromatic and heteroaromatic fulgides revealed significant advantages of the latter for applications as molecular switches and optical information storage systems [5,10]. These include higher thermal stability, longer lifetimes of the photocolored forms and higher yields of the photocoloration reactions and better persistance to photodegradation. It has been shown that annulation of benzene rings to the five-membered rings of pyrryl, thienyl and furanyl fulgides can promote the additional improvement of these properties [10,18–21]. In accordance with these data, we have previously synthesized and studied the photochromic properties of 2-benzo[*b*]thienylfulgides [22]. UV irradiation of acetonitrile solutions of these compounds results in the electrocyclic hexatriene–cyclohexadiene rearrangement of the ring-opened isomers **O** into the colored ring-closed ones **C** (Scheme 1) which exhibit enhanced spectral characteristics as compared with 2-thienyl and 3-benzo[*b*]thienyl analogues. Exposure of the colored solutions to the visible light leads to the reverse conversion into the primary colorless hexatriene form.

Here, we report the synthesis of 2-benzo[*b*]thienylfulgides with bulky diphenylmethylene substituents and the investigation of their photoinduced rearrangements.

## Results and Discussion

**Scheme 2:**
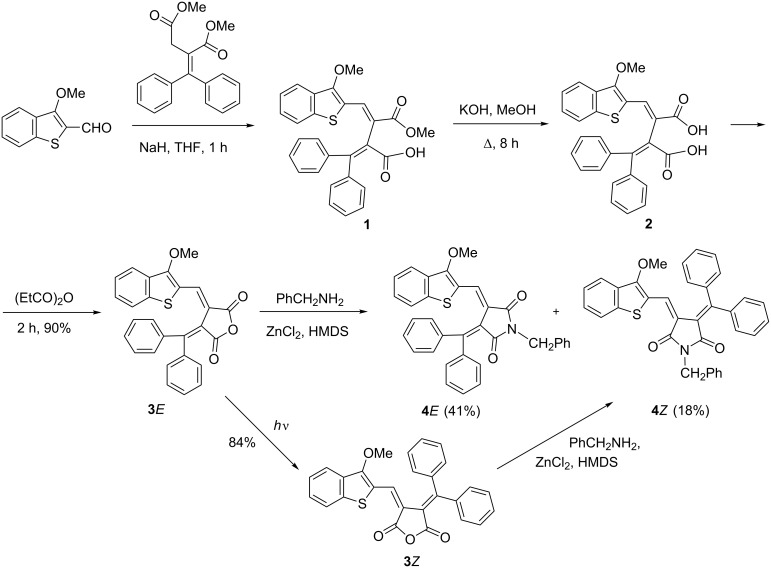
Synthesis of fulgides **3***E***, 3***Z* and fulgimides **4***E*, **4***Z*.

**Scheme 3:**
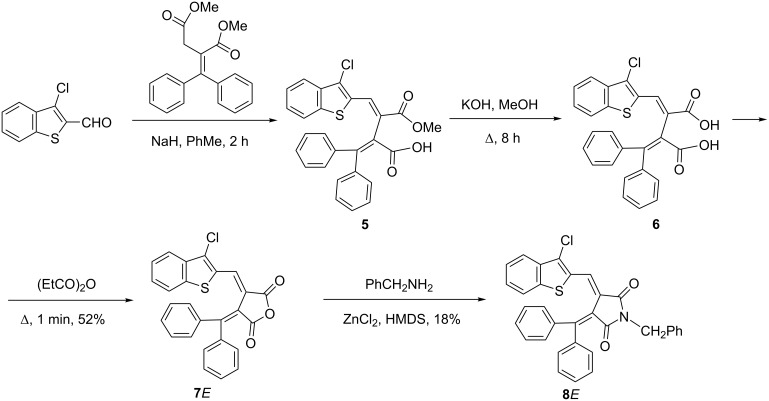
Synthesis of fulgide **7***E* and fulgimide **8***E*.

Fulgides **3***E***, 3***Z***, 7***E* and fulgimides **4***E*, **4***Z*, **8***E* were synthesized as shown in Scheme 2 and Scheme 3.

The Stobbe condensation of dimethyl 2-(diphenylmethylene)succinate with 1-methoxy- and 1-chlorobenzo[*b*]thiophene-2-carbaldehydes was used to obtain fulgides **3***E* and **7***E*, which led to the formation of intermediate monomethyl esters **1** and **5**. These esters were hydrolyzed by 10% KOH in methanol into the corresponding fulgenic acids **2** and **6**, which were purified and cyclized into **3***E* and **7***E* when treated with propionic anhydride. A special preparative method using a Sweko IP65 LED emitter was applied to convert the **3***E*-isomer into **3***Z* with a yield of 84%. The interaction of **3***E* with benzylamine in the presence of ZnCl_2_ and HMDS leads to the formation of a mixture of fulgimides **4***E* (41%) and **4***Z* (18%). Fulgides **3***Z* and **7***E* under similar conditions afford exclusively fulgimides **3***Z* and **7***E*, respectively, as the single products (Scheme 2 and Scheme 3).

According to IR, ^1^H and ^13^C NMR spectroscopy data *Z*- and *E*-isomers of fulgide **3** exist in the form of the open isomers **O**. This was also confirmed by X-ray diffraction study.

**Figure 1:**
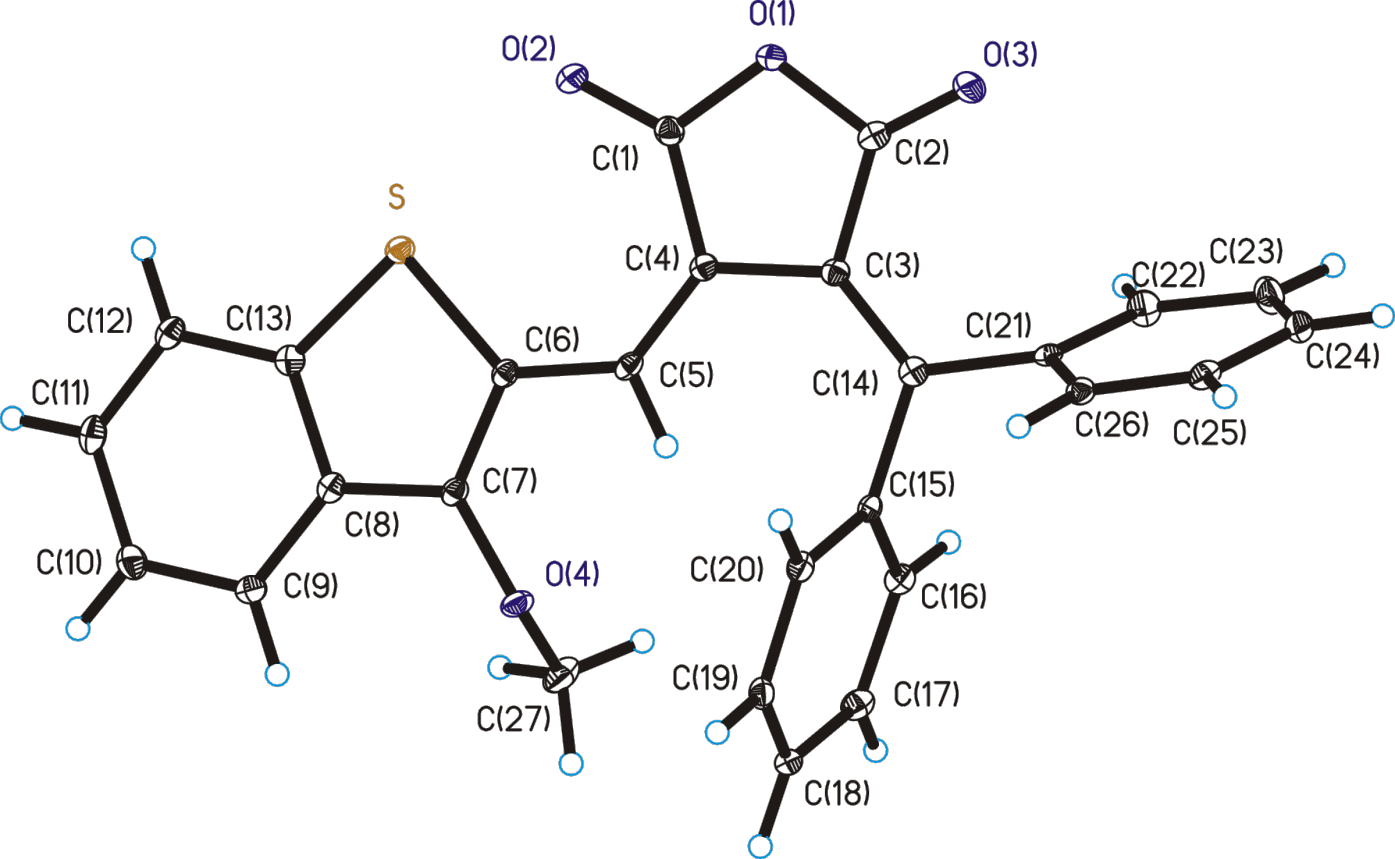
Molecular structure of **3***Z*. Thermal ellipsoids are drawn on the 30% probability level. Selected bond lengths (Å): C(3)–C(4) = 1.476(2), С(4)=С(5) = 1.360(2), С(3)=С(14) = 1.356(2).

**Figure 2:**
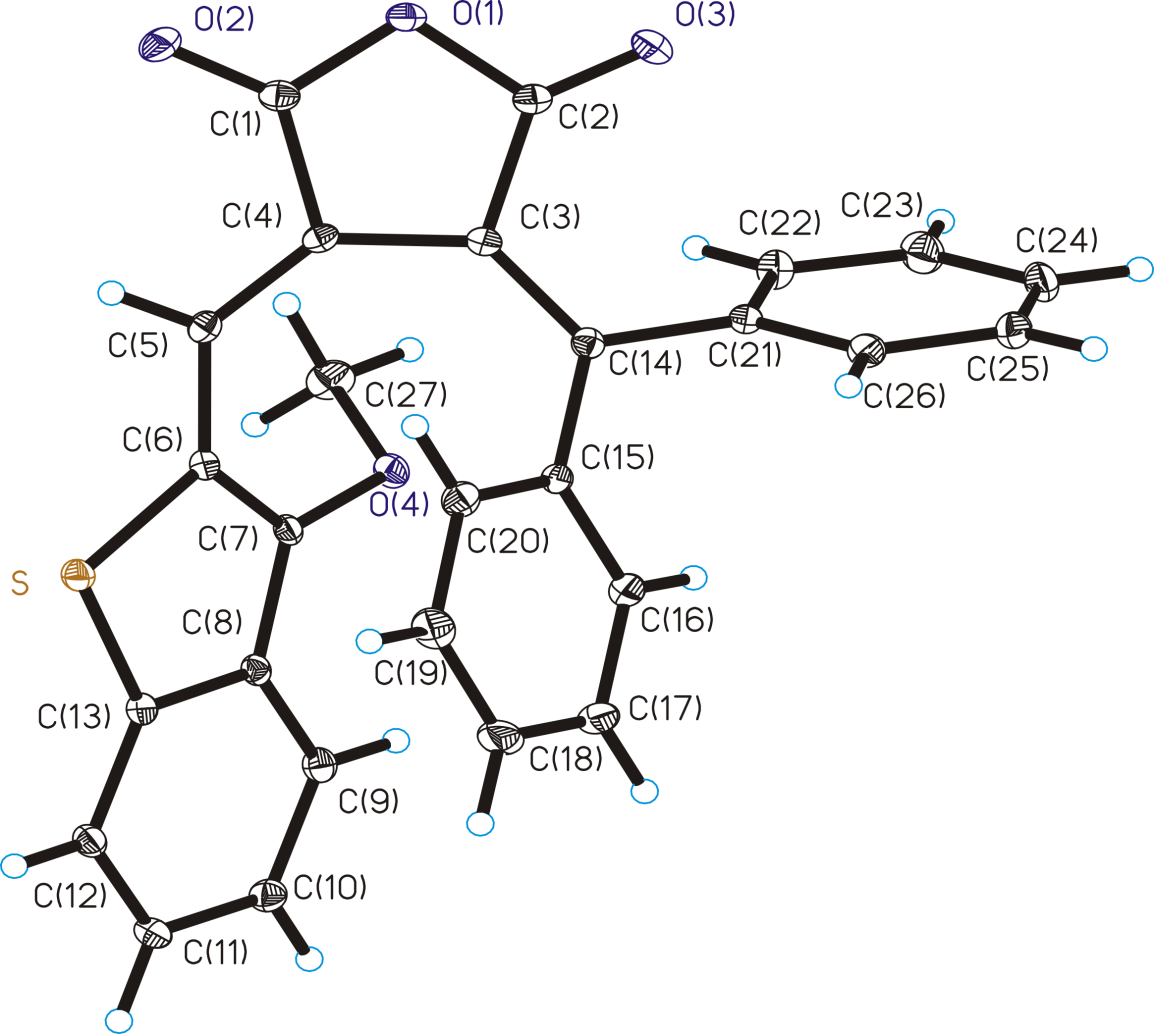
Molecular structure of **3***E*. Thermal ellipsoids are drawn on the 30% probability level. Selected bond lengths (Å): C(3)–C(4) = 1.461(2), С(4)=С(5) = 1.361(2), С(3)=С(14) = 1.375(2).

The molecular structures of fulgides **3***Z* and **3***E* are shown in Figure 1 and Figure 2.

Fulgide **3***Z* possesses *Z*-configuration at the double bond С(4)=С(5). The 3-methoxy-2-benzo[*b*]thiophene moiety in **3***Z* is planar and is almost coplanar with the planar furan-2,5-dione fragment (the dihedral angle is equal to 3.9°). The phenyl rings are rotated about the C–Ph bonds by respectively 84.9° (Ph(C(15)-C(20)) and 109.0° (Ph(C(21)-C(26)) relative to the furan-2,5-dione plane. The list of lengths and valence angles of **3***Z* is given in Table S1 (Supporting Information File 1).

Fulgide **3***E* has *E*-configuration at the double bond С(4)=С(5). The 3-methoxy-2-benzo[*b*]thiophene moiety in **3***E* is rotated relative to the furan-2,5-dione fragment by an angle of 36.5°. The torsion angle of the phenyl ring Ph(C(15)–C(20)) relative to the furan-2,5-dione plane (41.5°) is almost twice lesser than that in **3***Z*, whereas the torsion angle of the phenyl ring Ph(C(21)–C(26)) equal to 107.5° remains virtually unchanged. The bond lengths and valence angles of **3***E*, as well as the results of superimposition of **3*Z*** and **3*E*** by the atoms of furan-2,5-dione fragment O(1), O(2), O(3), C(1), C(2), C(3), C(4) (Scheme S1, Supporting Information File 1), are presented in Table S2 (Supporting Information File 1).

Fulgides **3** and **7** and fulgimides **4** and **8** in acetonitrile absorb in the range of 316–329 nm (S_2_←S_0_ transition) and 405–436 nm (S_1_←S_0_ transition) (Table 1). The transition S_1_←S_0_ band in the *E*-isomers is usually much more intense than the S_2_←S_0_ band, whereas in *Z*-isomers an inverse ratio of intensities is observed. *E*-isomers absorb at longer wavelengths than *Z*-isomers.

**Table 1:** Absorption maxima of the isomeric forms of fulgides **3** and **7,** and fulgimides **4** and **8** in acetonitrile^a^.

Compound	Ring-opened form **O**			
	*Z*-isomer		*E*-isomer	
	λ^abs^_max_, nm	ε_max_, L·mol^–1^·cm^−1^	λ^abs^_max_, nm	ε_max_, L·mol^–1^·cm^−1^

**3** **4** **7** **8**	327, 441 316, 414 – –	18.8, 11.4 16.8, 14.5 – –	326, 444 324, 416 329, 436 323, 432	12.0, 23.2 12.8, 23.4 15.6, 22.2 16.9, 15.6

^a^λ^abs^_max_, ε_max_ – the absorption maxima and the molar absorption coefficients.

**Scheme 4:**
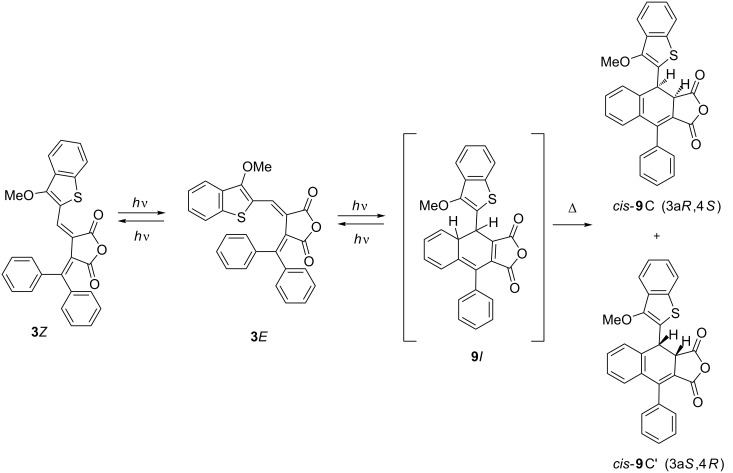
Photochemical rearrangements of fulgide **3***E* followed by1,5-H shift.

**Figure 3:**
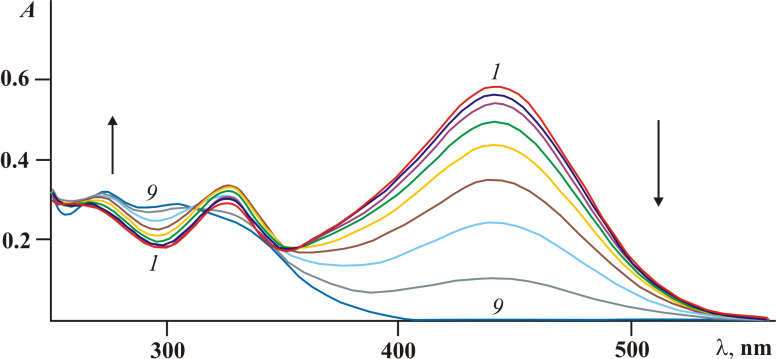
Electronic absorption spectra of fulgide **3***E* in acetonitrile solution before (*1*) and after irradiation with light of 365 nm for 5 (*2*), 10 (*3*), 20 (*4*), 40 (*5*), 60 (*6*), 80 (*7*), 120 (*8*) and 180 s (*9*) (2.5 × 10^−5^ M, *T* = 293 K).

Under irradiation in acetonitrile solution with light of 365 nm fulgide **3***E* manifest negative photoinduced properties [23] due to the rearrangement into 4,4a-dihydronaphtho[2,3-*c*]furan-1,3-dione **9***I* and then undergoing a 1,5-hydrogen shift with the formation of the colorless ring-closed isomer **9**C (Scheme 4, Figure 3).

However, the above described rearrangement drastically differs from those previously described due to its very high efficiency. The light-induced formation of the dihydronaphthalene core via 1,5-H shift has always been an extremely low-intensity process. Indeed, in the case of diphenylmethylene(aryl)fulgides the whole process (including the formation of a colored intermediate and its gradual decoloration) usually required 12–14 hours [14,17]. Even for the most similar in structure diphenylmethylene(indolyl)fulgide, the cyclization occurred only after extended irradiation (τ > 10^4^ s) [16], whereas for fulgides **3***Z* and **3***E* this time is at least two orders of magnitude less (Figure 3).

**9**C represents the racemate (racemic compound) (3a*R*,4*S*)/(3a*S*,4*R*)-4-(3-methoxybenzo[*b*]thiophen-2-yl)-9-phenyl-3a,4-dihydronaphtho[2,3-*c*]furan-1,3-dione with *cis*-configuration of hydrogen atoms C(4)H and C(5)H. Two doublet signals of these protons at 4.49 and 5.37 ppm with characteristic spin–spin coupling constant *J* = 7.3 Hz are observed in the ^1^H NMR spectrum [24]. The photoinduced transformation is thermally and photochemically irreversible.

**Figure 4:**
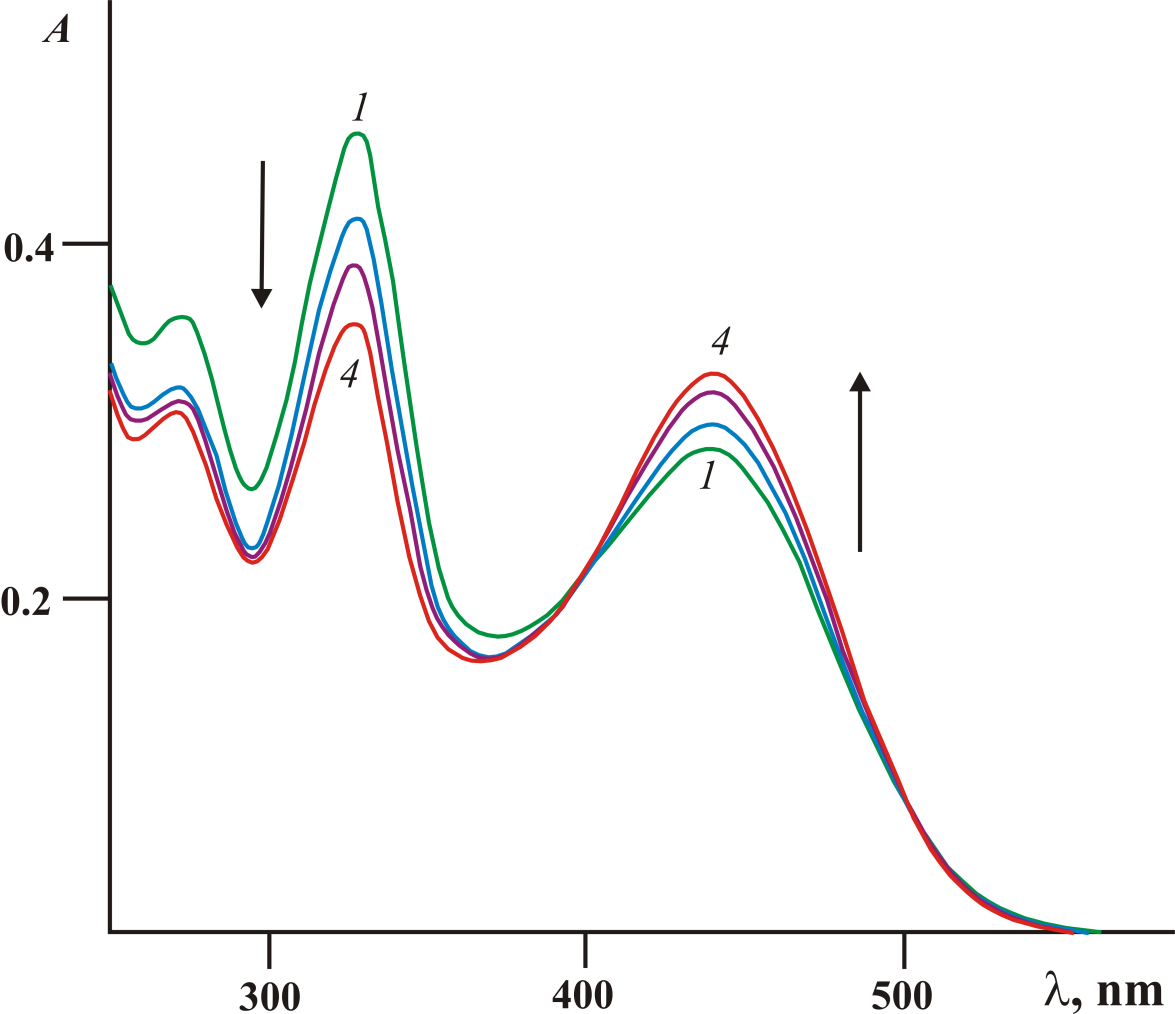
Electronic absorption spectra of fulgide **3***Z* in acetonitrile solution before (*1*) and after irradiation with light of 436 nm for 30 (*2*), 60 (*3*) and 100 (*4*) s (2.5 × 10^−5^ M, *T* = 293 K).

Although the appearance of **3***Z* and 4-(3-methoxybenzo[*b*]thiophen-2-yl)-9-phenyl-4,4a-dihydronaphtho[2,3-*c*]furan-1,3-dione (**9***I*) shown in Scheme 4 was not directly registered in the absorption spectra (Figure 3), their existence was evidenced by the data obtained in earlier studies [5–6,14–16]. Moreover, the occurrence of the *Z*/*E*-photoisomerization concomitant of the first stage of the rearrangement can be traced at small irradiation times (less than 1–2 min) of fulgide **3***Z* in acetonitrile solution with light of 436 nm. As shown in Figure 4 a slight bathochromic shift of the 441 nm band accompanied by a typical redistribution of intensity of absorption bands (see Table 1) is detected. At longer irradiation times, the rearrangement processes are reflected by the spectral curves depicted in Figure 3.

**Figure 5:**
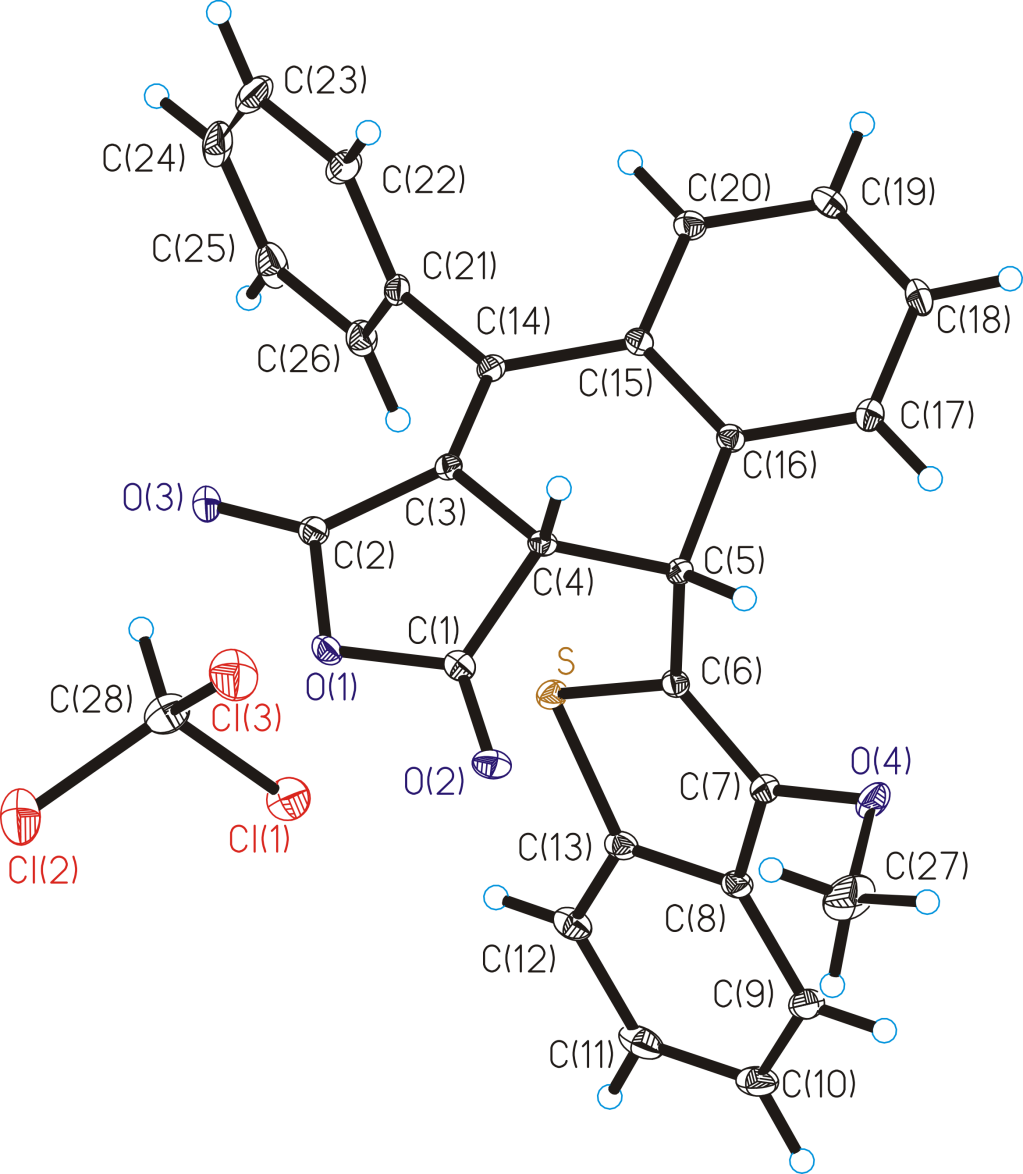
Molecular structure of photoproduct *cis*-**9**C’. Thermal ellipsoids are drawn on the 30% probability level. Selected bond lengths (Å): С(5)–С(16) = 1.531(3), С(15)=С(16) = 1.411(3), С(4)–С(5) = 1.535(3), C(3)–C(4) = 1.496(3), C(3)=C(14) = 1.349(3), С(14)–C(15) = 1.478(3).

Crystal packing of the product of the photoinduced rearrangement **9**C comprises alternating molecules of two enantiomers *cis*-**9**C and *cis*-**9**C’ corresponding to a racemic compound. The structure of *cis*-**9**C’ is shown in Figure 5. The dihydrobenzene cycle С(3)С(4)С(5)С(16)С(15)С(14) of *cis*-**9**C’ is nonplanar with inflections along the lines C(3)...C(16) and C(4)...C(16) at 15.0° and 44.4°, respectively. The C(4)–C(5) bond (its length is equal to 1.535(3) Å) is significantly elongated compared to the double C(4)–C(5) bond of **3***Z* (1.360(2) Å).

The list of other bond lengths and valence angles of *cis*-**9**C’ is presented in Table S3 (Supporting Information File 1).

**Scheme 5:**
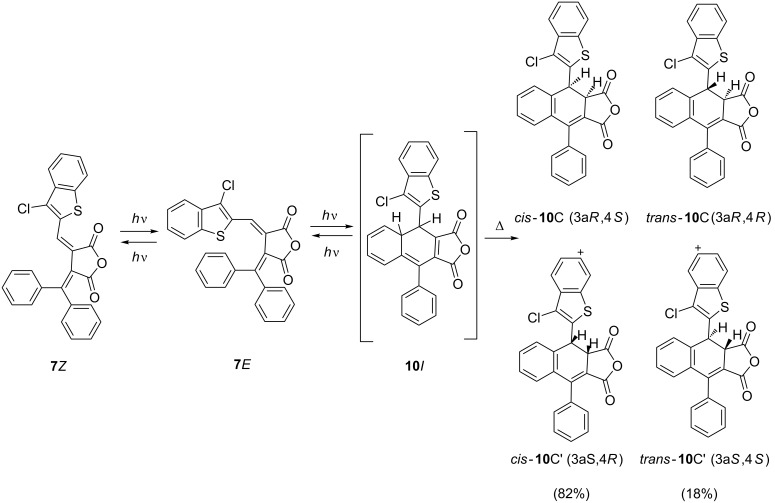
Photochemical rearrangements of fulgide **7***E* followed by1,5-H shift.

**Figure 6:**
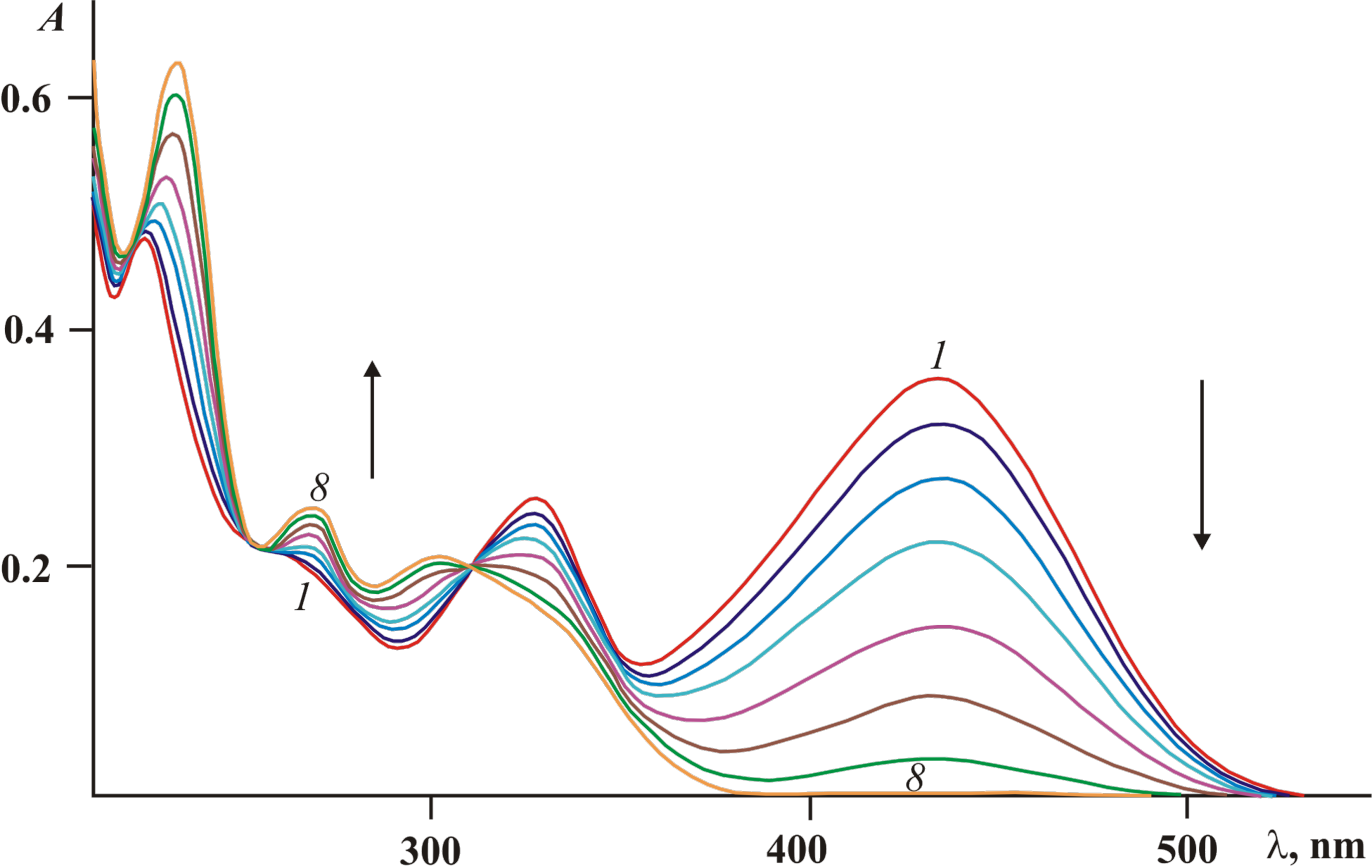
Electronic absorption spectra of fulgide **7***E* in acetonitrile solution before (*1*) and after irradiation with light of 365 nm for 5 (*2*), 10 (*3*), 20 (*4*), 40 (*5*), 80 (*6*), 120 (*7*) and 160 s (*8*) (2.5 × 10^−5^ M, *T* = 293 K).

Irradiation of an acetonitrile solution of fulgide **7***E* with light of 365 nm gives rise to its decoloration brought about by the formation of a mixture of ring-closed diastereomers *cis*-**10**C (82%) and *trans*-**10**C (18%) (Scheme 5, Figure 6). The ^1^H NMR spectra of **10**C contain signals of *cis*-3a,4-protons with a spin–spin coupling constant *J* = 7.3 Hz at 4.54 and 5.52 ppm, respectively, and of *trans*-3a,4-protons with a spin–spin coupling constant *J* = 15.4 Hz at 4.26 and 4.54 ppm, respectively.

**Scheme 6:**
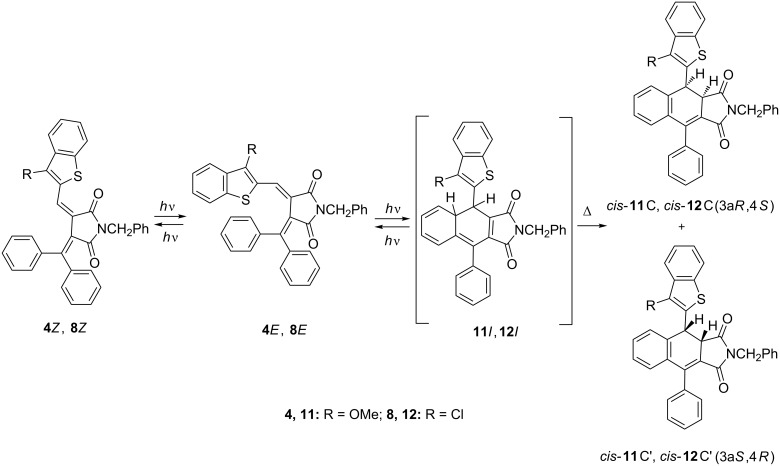
Photochemical rearrangements of fulgimides **4***E* and **8***E* followed by1,5-H shift.

**Figure 7:**
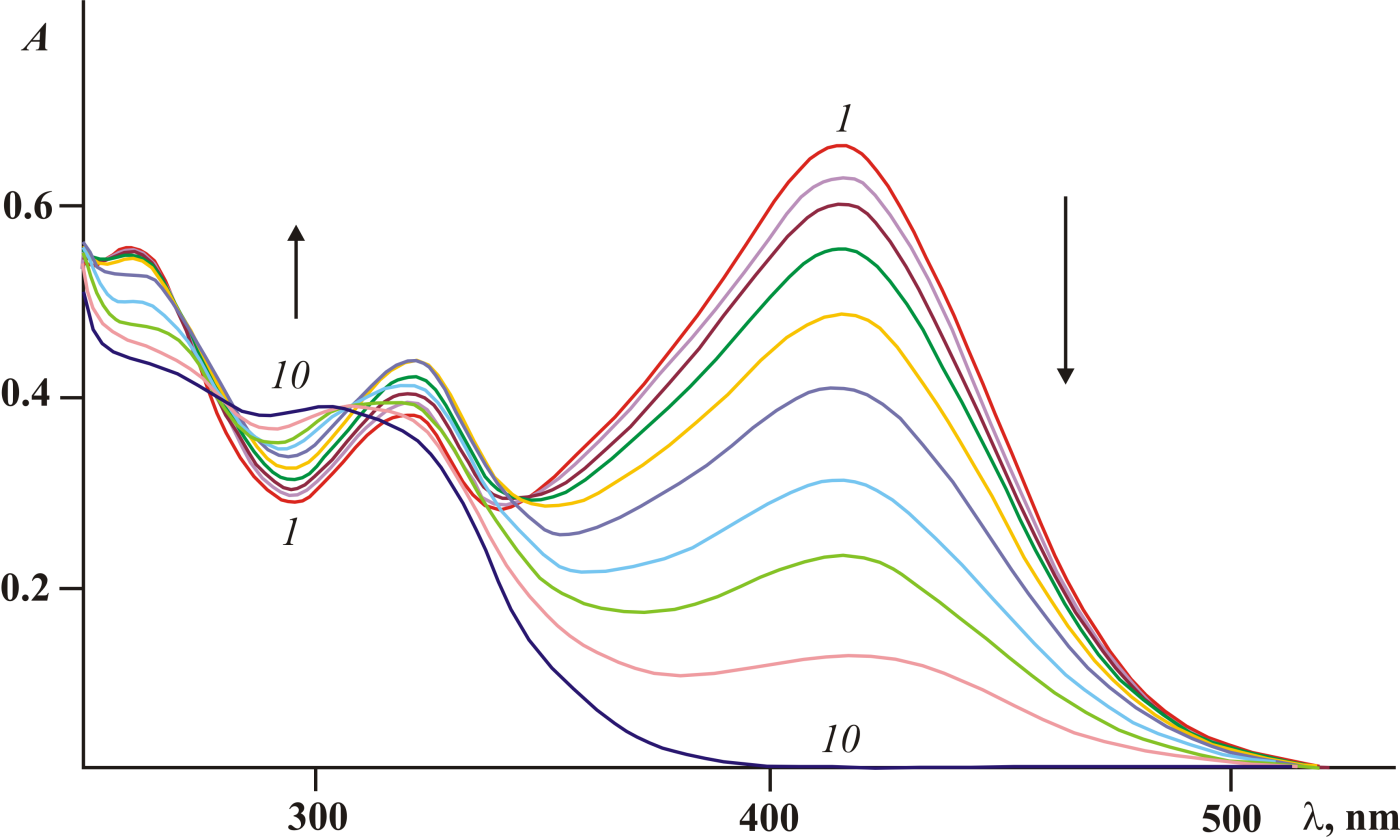
Electronic absorption spectra of fulgimide **8***E* in acetonitrile solution before (*1*) and after irradiation with light of 365 nm for 10 (*2*), 20 (*3*), 30 (*4*), 60 (*5*), 90 (*6*), 120 (*7*), 180 (*8*), 260 (*9*) and 320 s (*10*) (2.5 × 10^−5^ M, *T* = 293 K).

Similar to the initial fulgides, irradiation of acetonitrile solutions of fulgimides **4***E* and **8***E* with the light of 365 nm exhibits their negative photoinduced behavior caused by the formation of intermediate 4a,4-dihydro-1*H*-benzo[*f*]isoindol-1,3(2*H*)-diones **11***I* and **12***I* that rearrange in colorless ring-closed isomers **11**C and **12**C (Scheme 6**,** Figure 7). The observed transformations are thermally and photochemically irreversible.

To the best of our knowledge, this is the first example of an irreversible rearrangement of hetaryl fulgimide into colorless substituted dihydronaphthalene.

## Conclusion

To sum up, we report the synthesis of a series of fulgides possessing bulky diphenylmethylene substituents – 3-(diphenylmethylene)-4-((3-methoxybenzo[*b*]thiophen-2-yl)methylene)dihydrofuran-2,5-dione (*E*- and *Z*-isomers, **3**) and 3-((3-chlorobenzo[*b*]thiophen-2-yl)methylene)-4-diphenylmethylene)dihydrofuran-2,5-dione (*E*-isomer, **7**) and the corresponding fulgimides – 1-benzyl-3-(diphenylmethylene)-4-((3-methoxybenzo[*b*]thiophen-2-yl)methylene)pyrrolidine-2,5-dione (*E*- and *Z*-forms, **4**) and 1-benzyl-3-((3-chlorobenzo[*b*]thiophen-2-yl)methylene)-4-(diphenylmethylene)pyrrolidine-2,5-dione (*E*-form **8**)*.* In contrast to the compounds with less voluminous dimethyl methylene groups, which under irradiation in solution formed deeply colored ring-closed forms, the obtained fulgides/fulgimides after exposure to light of 365 nm irreversibly rearrange into the colorless closed isomers, 3a,4-dihydronaphtho[2,3-*c*]furan-1,3-diones **9** and **10** and 3a,4-dihydro-1*H*-benzo[*f*]isoindol-1,3(2*H*)-diones **11** and **12**. These are formed due to the symmetry-allowed excited state electrocyclic reaction followed by the thermally induced 1,5-hydrogen shift reaction. This transformation represents a highly efficient process in comparison with the similar low-efficiency rearrangements of known diphenylmethylene(aryl(hetaryl))fulgides. The key structures of these light-induced reactions – *E*-, *Z*-isomers and closed forms **C** were identified with the use of ^1^H and ^13^C NMR spectroscopy and their molecular structures confirmed by X-ray diffraction studies.

## Experimental

**General:** The ^1^H and ^13^C NMR spectra in CDCl_3_ were obtained on an integrated analytical LC-SPE-NMR-MS AVANCE-600 system from Bruker (600 MHz, 151 MHz for ^13^C) The signals were referred with respect to the signals of residual protons of deuterated solvent (7.24 ppm). The IR spectra were recorded on a Varian Excalibur 3100 FTIR instrument using the attenuated total internal reflection technique (ZnSe crystal). The electronic absorption spectra were recorded on a Varian Cary 100 spectrophotometer. The irradiation of acetonitrile solutions in quartz cells (*l* = 10 mm, *V* = 2**^.^**mL) with a high pressure Hg lamp (250 W) was performed on a Newport 66941 equipment supplied with a set of interferential light filters. The intensity of light was 3.3·10^16^ photons·s^−1^ for the spectral line 365 nm. For preparative purposes a Sweko IP65 (SUL-S1-20W-230-4000K-WH) LED emitter was exploited. Acetonitrile of spectroscopic grade (Aldrich) was used to prepare solutions. Melting points were determined on a PTP (M) instrument.

**X-ray diffraction study:** The X-ray diffraction studies of crystals of **3***Z***, 3***E* and **9** were performed with a Bruker XSCANS device (λ(Мо Кα) = 0.71073 Å, \w scans). Bond lengths and valence angles of **3***Z***, 3***E* and **9** are presented in Tables S1–S3 (Supporting Information File 1). The main crystallographic data and characteristics are in Table S4 (Supporting Information File 1). The semi-empirical absorption correction based on equivalent reflections was applied. The structure was solved by direct methods [25] and refined by full-matrix least-squares technique on F^2^ with anisotropic displacement parameters for non-hydrogen atoms. The hydrogen atoms were localized in the difference-Fourier map and included in the refinement with fixed positional (riding model) and isotropic displacement parameters. All calculations were carried out using the SHELXTL program suite [26]. CCDC 1951883, 1951883 and 1951885 contain the supplementary crystallographic data for the **3***Z***, 3***E* and **9**, respectively. These data can be obtained free of charge via http://www.ccdc.cam.ac.uk/conts/retrieving.html, or from the Cambridge Crystallographic Data Centre, 12 Union Road, Cambridge CB2 1EZ, UK; fax: (+44) 1223-336-033; or e-mail: deposit@ccdc.cam.ac.uk.

## Supporting Information

File 1 X-ray analysis data of **3***Z*, **3***E* and **9***C*.

File 2 ^1^H, ^13^C NMR and IR spectra of all novel compounds.

File 3 Experimental procedures and characterization data for fulgides **3***E*, **3***Z*, **7***E*, fulgimides **4***Z*, **4***E*, **8***E* and photo products **9**C, **10**C, **11**C and **12**C.
