# Supplementary material for: Synthesis and highly efficient light-induced rearrangements of diphenylmethylene(2-benzo[b]thienyl)fulgides and fulgimides
Source: Beilstein J Org Chem. 2020 Jul 22;16:1820–9. doi: 10.3762/bjoc.16.149 (PMC7385394; doi:10.3762/bjoc.16.149)
Supplement: File 1 — X-ray analysis data of 3Z, 3E and 9C. [file Beilstein_J_Org_Chem-16-1820-s001.pdf]

BEILSTEIN JOURNAL OF ORGANIC CHEMISTRY

Supporting Information

for

Synthesis and highly efficient light-induced rearrangements of diphenylmethylene(2-benzo[*b*]thienyl)fulgides and fulgimides

Vladimir P. Rybalkin, Sofiya Yu. Zmeeva, Lidiya L. Popova, Valerii V. Tkachev, Andrey N. Utenyshev, Olga Yu. Karlutova, Alexander D. Dubonosov, Vladimir A. Bren, Sergey M. Aldoshin and Vladimir I. Minkin

Beilstein J. Org. Chem. **2020,** *16,* 1820–1829. doi:10.3762/bjoc.16.149

X-ray analysis data of 3*Z*, 3*E* and 9*C*

License and Terms: This is a supporting information file under the terms of the Creative Commons Attribution License (http://creativecommons.org/licenses/by/4.0). Please note that the reuse, redistribution and reproduction in particular requires that the authors and source are credited.
The license is subject to the *Beilstein Journal of Organic Chemistry* terms and conditions: (https://www.beilstein-journals.org/bjoc)

Contents

1. Table S1. Bond lengths [A] and angles [deg] for **3***Z*……………………………….S2

2. Table S2. Bond lengths [A] and angles [deg] for **3***E*………………………………..S4

3. Table S3. Bond lengths [A] and angles [deg] for **9**C………………………………..S6

4. Table S4. Crystal data and structure refinement for **3***Z***, 3***E* and **9**C………………S9

5. Scheme S1. The superimposed molecules **3***Z* and **3***E*……………………………S11

Table S1. Bond lengths [A] and angles [deg] for **3***Z*.

S-C(13)	1.7406(19)
S-C(6)	1.7622(18)
O(1)-C(1)	1.385(2)
O(1)-C(2)	1.404(2)
O(2)-C(1)	1.202(2)
O(3)-C(2)	1.192(2)
O(4)-C(7)	1.368(2)
O(4)-C(27)	1.441(2)
C(1)-C(4)	1.467(3)
C(2)-C(3)	1.491(3)
C(3)-C(14)	1.356(2)
C(3)-C(4)	1.476(2)
C(4)-C(5)	1.360(2)
C(5)-C(6)	1.433(2)
C(6)-C(7)	1.384(2)
C(7)-C(8)	1.428(2)
C(8)-C(9)	1.404(2)
C(8)-C(13)	1.406(2)
C(9)-C(10)	1.383(2)
C(10)-C(11)	1.399(3)
C(11)-C(12)	1.383(3)
C(12)-C(13)	1.397(2)
C(14)-C(21)	1.496(2)
C(14)-C(15)	1.501(2)
C(15)-C(20)	1.395(2)
C(15)-C(16)	1.398(2)
C(16)-C(17)	1.386(3)
C(17)-C(18)	1.386(3)
C(18)-C(19)	1.393(3)
C(19)-C(20)	1.383(3)
C(21)-C(26)	1.394(2)
C(21)-C(22)	1.394(3)
C(22)-C(23)	1.390(3)
C(23)-C(24)	1.386(3)
C(24)-C(25)	1.386(3)
C(25)-C(26)	1.387(2)
C(13)-S-C(6)	91.59(9)
C(1)-O(1)-C(2)	110.67(14)
C(7)-O(4)-C(27)	114.85(14)
O(2)-C(1)-O(1)	119.25(16)
O(2)-C(1)-C(4)	131.49(18)
O(1)-C(1)-C(4)	109.26(15)
O(3)-C(2)-O(1)	118.90(16)
O(3)-C(2)-C(3)	133.10(17)
O(1)-C(2)-C(3)	107.98(15)
C(14)-C(3)-C(4)	132.17(16)
C(14)-C(3)-C(2)	122.07(16)

C(4)-C(3)-C(2)	105.75(15)
C(5)-C(4)-C(1)	125.19(17)
C(5)-C(4)-C(3)	128.42(17)
C(1)-C(4)-C(3)	106.34(15)
C(4)-C(5)-C(6)	133.77(17)
C(7)-C(6)-C(5)	119.66(16)
C(7)-C(6)-S	110.31(13)
C(5)-C(6)-S	129.99(14)
O(4)-C(7)-C(6)	121.89(16)
O(4)-C(7)-C(8)	123.21(16)
C(6)-C(7)-C(8)	114.85(16)
C(9)-C(8)-C(13)	120.00(16)
C(9)-C(8)-C(7)	129.07(17)
C(13)-C(8)-C(7)	110.88(16)
C(10)-C(9)-C(8)	118.86(17)
C(9)-C(10)-C(11)	120.43(18)
C(12)-C(11)-C(10)	121.68(17)
C(11)-C(12)-C(13)	118.04(17)
C(12)-C(13)-C(8)	120.97(17)
C(12)-C(13)-S	126.64(15)
C(8)-C(13)-S	112.34(13)
C(3)-C(14)-C(21)	123.43(16)
C(3)-C(14)-C(15)	121.68(16)
C(21)-C(14)-C(15)	114.89(15)
C(20)-C(15)-C(16)	119.06(17)
C(20)-C(15)-C(14)	121.17(16)
C(16)-C(15)-C(14)	119.77(16)
C(17)-C(16)-C(15)	120.19(18)
C(16)-C(17)-C(18)	120.41(17)
C(17)-C(18)-C(19)	119.65(17)
C(20)-C(19)-C(18)	120.12(18)
C(19)-C(20)-C(15)	120.51(17)
C(26)-C(21)-C(22)	118.72(17)
C(26)-C(21)-C(14)	119.92(16)
C(22)-C(21)-C(14)	121.31(17)
C(23)-C(22)-C(21)	120.42(18)
C(24)-C(23)-C(22)	120.33(18)
C(25)-C(24)-C(23)	119.54(18)
C(24)-C(25)-C(26)	120.22(18)
C(25)-C(26)-C(21)	120.69(17)

Table S2. Bond lengths [A] and angles [deg] for **3***E*.

S-C(13)	1.7363(16)
S-C(6)	1.7562(15)
O(1)-C(1)	1.3848(18)
O(1)-C(2)	1.4092(18)
O(2)-C(1)	1.1987(18)
O(3)-C(2)	1.1905(18)
O(4)-C(7)	1.3678(16)
O(4)-C(27)	1.4354(18)
C(1)-C(4)	1.480(2)
C(2)-C(3)	1.487(2)
C(3)-C(14)	1.375(2)
C(3)-C(4)	1.461(2)
C(4)-C(5)	1.361(2)
C(5)-C(6)	1.432(2)
C(6)-C(7)	1.375(2)
C(7)-C(8)	1.432(2)
C(8)-C(9)	1.401(2)
C(8)-C(13)	1.4080(19)
C(9)-C(10)	1.382(2)
C(10)-C(11)	1.408(2)
C(11)-C(12)	1.384(2)
C(12)-C(13)	1.398(2)
C(14)-C(15)	1.472(2)
C(14)-C(21)	1.492(2)
C(15)-C(20)	1.399(2)
C(15)-C(16)	1.403(2)
C(16)-C(17)	1.386(2)
C(17)-C(18)	1.388(3)
C(18)-C(19)	1.384(3)
C(19)-C(20)	1.393(2)
C(21)-C(22)	1.393(2)
C(21)-C(26)	1.394(2)
C(22)-C(23)	1.386(2)
C(23)-C(24)	1.384(3)
C(24)-C(25)	1.384(3)
C(25)-C(26)	1.390(2)
C(13)-S-C(6)	91.72(7)
C(1)-O(1)-C(2)	109.83(12)
C(7)-O(4)-C(27)	115.42(11)
O(2)-C(1)-O(1)	120.03(14)
O(2)-C(1)-C(4)	130.81(15)
O(1)-C(1)-C(4)	109.14(12)
O(3)-C(2)-O(1)	118.29(14)
O(3)-C(2)-C(3)	133.00(14)
O(1)-C(2)-C(3)	108.59(12)
C(14)-C(3)-C(4)	132.88(14)
C(14)-C(3)-C(2)	120.87(13)

C(4)-C(3)-C(2)	105.70(12)
C(5)-C(4)-C(3)	137.05(14)
C(5)-C(4)-C(1)	116.34(13)
C(3)-C(4)-C(1)	106.42(13)
C(4)-C(5)-C(6)	129.69(14)
C(7)-C(6)-C(5)	130.02(14)
C(7)-C(6)-S	111.06(11)
C(5)-C(6)-S	118.89(11)
O(4)-C(7)-C(6)	125.45(14)
O(4)-C(7)-C(8)	120.56(13)
C(6)-C(7)-C(8)	113.98(13)
C(9)-C(8)-C(13)	119.56(14)
C(9)-C(8)-C(7)	129.00(13)
C(13)-C(8)-C(7)	111.43(13)
C(10)-C(9)-C(8)	119.27(14
C(9)-C(10)-C(11)	120.56(15)
C(12)-C(11)-C(10)	121.12(15)
C(11)-C(12)-C(13)	118.14(14)
C(12)-C(13)-C(8)	121.35(14)
C(12)-C(13)-S	126.85(12)
C(8)-C(13)-S	111.79(11)
C(3)-C(14)-C(15)	124.38(13)
C(3)-C(14)-C(21)	120.12(13)
C(15)-C(14)-C(21)	115.48(12)
C(20)-C(15)-C(16)	118.60(14)
C(20)-C(15)-C(14)	121.34(14)
C(16)-C(15)-C(14)	120.04(14)
C(17)-C(16)-C(15)	120.65(16)
C(16)-C(17)-C(18)	120.15(17)
C(19)-C(18)-C(17)	119.89(16
C(18)-C(19)-C(20)	120.37(17)
C(19)-C(20)-C(15)	120.32(16)
C(22)-C(21)-C(26)	118.76(14)
C(22)-C(21)-C(14)	120.87(13)
C(26)-C(21)-C(14)	120.32(14)
C(23)-C(22)-C(21)	120.68(15)
C(24)-C(23)-C(22)	120.01(17)
C(25)-C(24)-C(23)	120.05(15)
C(24)-C(25)-C(26)	119.96(16)
C(25)-C(26)-C(21)	120.50(16)

Table S3. Bond lengths [A] and angles [deg] for **9**C.

S-C(13)	1.743(2)
S-C(6)	1.7473(19)
O(1)-C(1)	1.390(2)
O(1)-C(2)	1.411(2)
O(2)-C(1)	1.193(2)
O(3)-C(2)	1.190(2)
O(4)-C(7)	1.373(2)
O(4)-C(27)	1.436(3)
C(1)-C(4)	1.510(3)
C(2)-C(3)	1.473(3)
C(3)-C(14)	1.349(3)
C(3)-C(4)	1.496(3)
C(4)-C(5)	1.535(3)
C(5)-C(6)	1.504(3)
C(5)-C(16)	1.531(3)
C(6)-C(7)	1.363(3)
C(7)-C(8)	1.440(3)
C(8)-C(13)	1.405(3)
C(8)-C(9)	1.410(3)
C(9)-C(10)	1.378(3)
C(10)-C(11)	1.396(4)
C(11)-C(12)	1.383(3)
C(12)-C(13)	1.399(3)
C(14)-C(15)	1.478(3)
C(14)-C(21)	1.490(3)
C(15)-C(20)	1.398(3)
C(15)-C(16)	1.411(3)
C(16)-C(17)	1.390(3)
C(17)-C(18)	1.394(3)
C(18)-C(19)	1.389(3)
C(19)-C(20)	1.393(3)
C(21)-C(26)	1.388(3)
C(21)-C(22)	1.392(3)
C(22)-C(23)	1.389(3)
C(23)-C(24)	1.384(4)
C(24)-C(25)	1.384(4)
C(25)-C(26)	1.394(3)
C(28)-Cl(21)	1.715(7)
C(28)-Cl(11)	1.750(8)
C(28)-Cl(31)	1.751(8)
C(28)-Cl(1)	1.758(2)
C(28)-Cl(3)	1.763(2)
C(28)-Cl(2)	1.776(3)
C(13)-S-C(6)	91.61(10)
C(1)-O(1)-C(2)	111.18(14)
C(7)-O(4)-C(27)	112.58(18)
O(2)-C(1)-O(1)	120.69(17)

O(2)-C(1)-C(4)	129.77(18)
O(1)-C(1)-C(4)	109.52(16)
O(3)-C(2)-O(1)	119.67(17)
O(3)-C(2)-C(3)	133.11(18)
O(1)-C(2)-C(3)	107.17(15)
C(14)-C(3)-C(2)	128.19(17)
C(14)-C(3)-C(4)	122.76(17)
C(2)-C(3)-C(4)	108.48(16)
C(3)-C(4)-C(1)	102.88(15)
C(3)-C(4)-C(5)	111.77(15)
C(1)-C(4)-C(5)	116.66(16)
C(6)-C(5)-C(16)	111.61(16)
C(6)-C(5)-C(4)	113.97(15)
C(16)-C(5)-C(4)	107.99(15)
C(7)-C(6)-C(5)	124.99(17)
C(7)-C(6)-S	111.49(15)
C(5)-C(6)-S	123.52(14)
C(6)-C(7)-O(4)	122.22(18)
C(6)-C(7)-C(8)	114.19(18)
O(4)-C(7)-C(8)	123.58(17)
C(13)-C(8)-C(9)	119.46(19)
C(13)-C(8)-C(7)	110.96(17)
C(9)-C(8)-C(7)	129.6(2)
C(10)-C(9)-C(8)	118.6(2)
C(9)-C(10)-C(11)	121.2(2)
C(12)-C(11)-C(10)	121.4(2)
C(11)-C(12)-C(13)	117.7(2)
C(12)-C(13)-C(8)	121.56(19)
C(12)-C(13)-S	126.70(17)
C(8)-C(13)-S	111.74(15)
C(3)-C(14)-C(15)	117.65(17)
C(3)-C(14)-C(21)	122.66(17)
C(15)-C(14)-C(21)	119.67(16)
C(20)-C(15)-C(16)	119.08(17)
C(20)-C(15)-C(14)	121.28(17)
C(16)-C(15)-C(14)	119.58(17)
C(17)-C(16)-C(15)	119.63(18)
C(17)-C(16)-C(5)	119.41(17)
C(15)-C(16)-C(5)	120.89(16)
C(16)-C(17)-C(18)	120.65(19)
C(19)-C(18)-C(17)	120.06(18)
C(18)-C(19)-C(20)	119.72(19)
C(19)-C(20)-C(15)	120.85(19)
C(26)-C(21)-C(22)	119.39(19)
C(26)-C(21)-C(14)	120.53(18)
C(22)-C(21)-C(14)	120.09(19)
C(23)-C(22)-C(21)	120.0(2)
C(24)-C(23)-C(22)	120.5(2)
C(25)-C(24)-C(23)	119.7(2)
C(24)-C(25)-C(26)	120.1(2)

C(21)-C(26)-C(25)	120.3(2)
Cl(21)-C(28)-Cl(11)	142.0(7)
Cl(21)-C(28)-Cl(31)	116.8(8)
Cl(11)-C(28)-Cl(31)	101.0(8)
Cl(21)-C(28)-Cl(1)	122.9(5)
Cl(11)-C(28)-Cl(1)	21.8(5)
Cl(31)-C(28)-Cl(1)	119.5(7)
Cl(21)-C(28)-Cl(3)	117.8(4)
Cl(11)-C(28)-Cl(3)	98.1(5)
Cl(31)-C(28)-Cl(3)	22.5(6)
Cl(1)-C(28)-Cl(3)	110.77(13)
Cl(21)-C(28)-Cl(2)	27.0(5)
Cl(11)-C(28)-Cl(2)	131.8(6)
Cl(31)-C(28)-Cl(2)	119.2(7)
Cl(1)-C(28)-Cl(2)	110.09(14)
Cl(3)-C(28)-Cl(2)	108.93(13)

Table S4. Crystal data and structure refinement for **3*Z*, 3*E*** and **9C.**

Compound	**3*Z***	**3*E***	**9C**
Empirical formula	$C_{27}H_{18}O_4S$	$C_{27}H_{18}O_4S$	$C_{27}H_{19}Cl_3O_4S$
Formula weight	438.47	438.47	557.84
Temperature, K	100.0(1)	100.0(1)	100.0(1)
Wavelength, Å	0.71073	0.71073	0.71073
Crystal system, space group	Monoclinic, P 2_1/c	Triclinic, P -1	Monoclinic, P 2_1/c
Volume, Å^3	2071.31(9)	1060.57(10)	2468.0(9)
Unit cell dimensions, Å and deg.	a = 11.0297(3) α = 90°, b = 7.46108(18) β = 97.289(3)°, c = 25.3750(7) γ = 90°	a = 9.8735(6) α= 69.581(5)°, b = 10.5094(6) β= 87.765(5)°, c =11.2616(6) γ= 75.859(5)°	a = 13.170(3) α = 90°, b = 9.6300(19) β=104.50(3)°, c = 20.100(4) γ = 90°
Z, Calculated density, g/cm^3	4, 1.406	2, 1.373	4, 1.501
Absorption coefficient, mm^{-1}	0.190	0.186	0.491
F(000)	912	456	1144
Crystal size, mm	0.36 x 0.35 x 0.33	0.48 x 0.44 x 0.41	0.50 x 0.15 x 0.05
Theta range for data collection, deg.	3.17 to 26.07	2.85 to 29.07	2.98 to 29.07

Limiting indices	-13<=h<=8, 31<=l<=31, -8<=k<=9	-12<=h<=13, -14<=k<=12, -15<=l<=15	-17<=h<=18, -12<=k<=13, -27<=l<=19
Reflections collected / unique	8306/4088[R(int)=0.0311]	9115 / 5666 [R(int) = 0.0244]	22780 / 6554 [R(int) = 0.0335]
Completeness to theta	26.07° 99.5 %	29.07° 99.9%	29.07 99.4 %
Absorption correction	Semi-empirical from equivalents	Semi-empirical from equivalents	Semi-empirical from equivalents
Max. and min. transmission	1.000 and 0.9893	1.0000 and 0.9690	1.0000 and 0.92002
Data / restraints / parameters	4088 / 0 / 289	5666 / 0 / 289	6554 / 33 / 335
Goodness-of-fit on F^2	0.986	1.026	1.034
Final R indices [I>2sigma(I)]	R1 = 0.0413, wR2 = 0.0782	R1 = 0.0434, wR2 = 0.0934	R1 = 0.0441, wR2 = 0.1054
R indices (all data)	R1 = 0.0634, wR2 = 0.0852	R1 = 0.0605, wR2 = 0.1044	R1 = 0.0605, wR2 = 0.1134
Largest diff. peak and hole, e.$Å^{-3}$	0.332 and -0.315	0.377 and -0.350	0.710 and -0.792

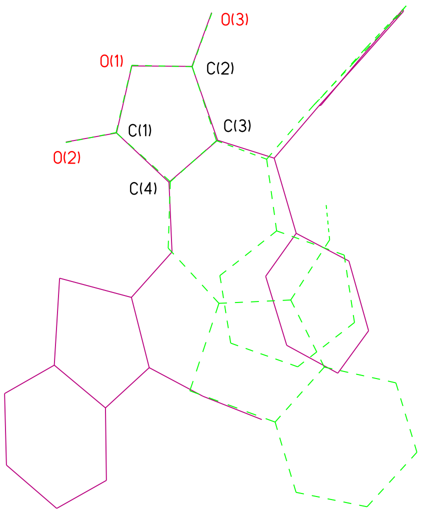

Scheme S1. The superimposed molecules **3***Z* and **3***E* by the atoms of furan-2,5-dione fragment O(1), O(2), O(3), C(1), C(2), C(3), C(4) (the solid line corresponds to **3***Z*, the dotted line - to **3***E*).

Crystals formed by **3***Z* have a more favorable lattice energy, compared to **3***E*, equal to -51.5 kcal/mol (for a **3***E* crystal, the lattice energy is −43.8 kcal/mol). In the crystal structure of **3***Z* there are contacts between sulfur atoms S...S*: 3.853 (−x, 1−y, −z), 4.411 (−x, −y, −z), 7.461 (−x, −1−y, z and −x, 1−y, −z). In the crystal structure of **3***E* there is a single contact S...S*: 6.892 (2−x, 2−y, −z).
