# Supplementary material for: Synthesis and highly efficient light-induced rearrangements of diphenylmethylene(2-benzo[b]thienyl)fulgides and fulgimides
Source: Beilstein J Org Chem. 2020 Jul 22;16:1820–9. doi: 10.3762/bjoc.16.149 (PMC7385394; doi:10.3762/bjoc.16.149)

BEILSTEIN JOURNAL OF ORGANIC CHEMISTRY

Supporting Information

for

Synthesis and highly efficient light-induced rearrangements of diphenylmethylene(2-benzo[*b*]thienyl)fulgides and fulgimides

Vladimir P. Rybalkin, Sofiya Yu. Zmeeva, Lidiya L. Popova, Valerii V. Tkachev, Andrey N. Utenyshev, Olga Yu. Karlutova, Alexander D. Dubonosov, Vladimir A. Bren, Sergey M. Aldoshin and Vladimir I. Minkin

Beilstein J. Org. Chem. **2020,** *16,* 1820–1829. doi:10.3762/bjoc.16.149

^{1}H, ^{13}C NMR and IR spectra of all novel compounds

License and Terms: This is a supporting information file under the terms of the Creative Commons Attribution License (http://creativecommons.org/licenses/by/4.0). Please note that the reuse, redistribution and reproduction in particular requires that the authors and source are credited.
The license is subject to the *Beilstein Journal of Organic Chemistry* terms and conditions: (https://www.beilstein-journals.org/bjoc)

Contents

1. ^{1}H NMR spectra..S2

2. ^{13}C NMR spectra………………………………………………………………….....S11

3. IR spectra…………………………………………………………………………S20

Figure S1: The ^{1}H NMR spectrum of **3***E* in $CDCl_3$.

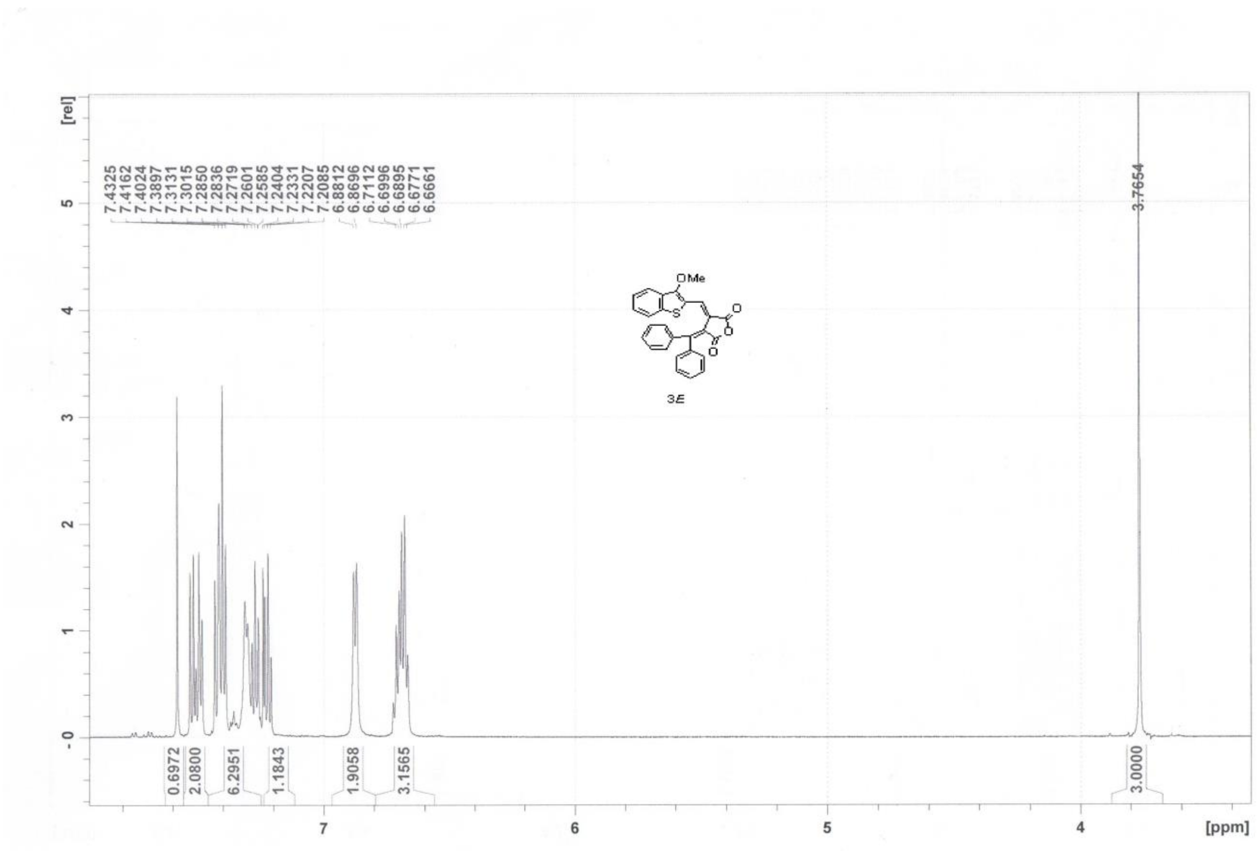

Figure S2: The ^{1}H NMR spectrum of **3***Z* in $CDCl_3$.

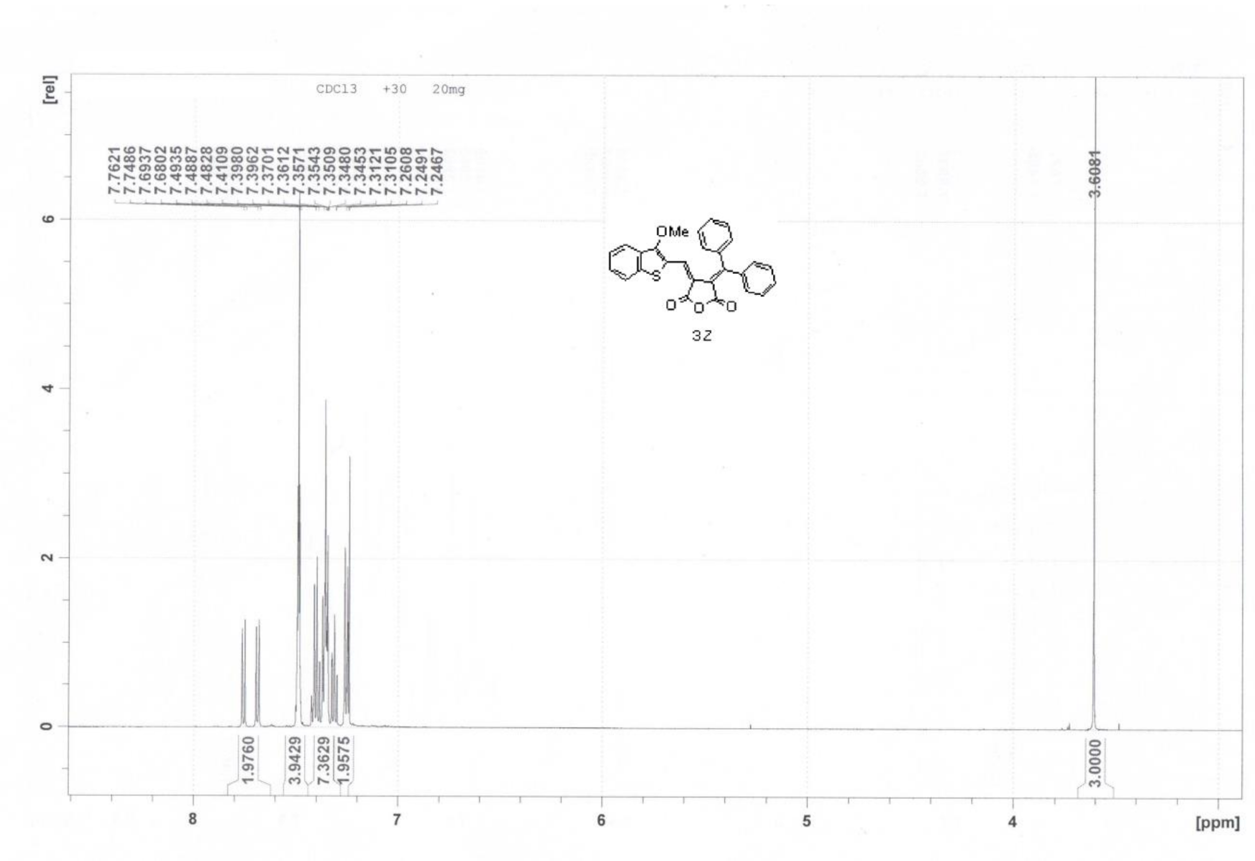

Figure S3: The ^1H NMR spectrum of **9**C in $CDCl_3$.

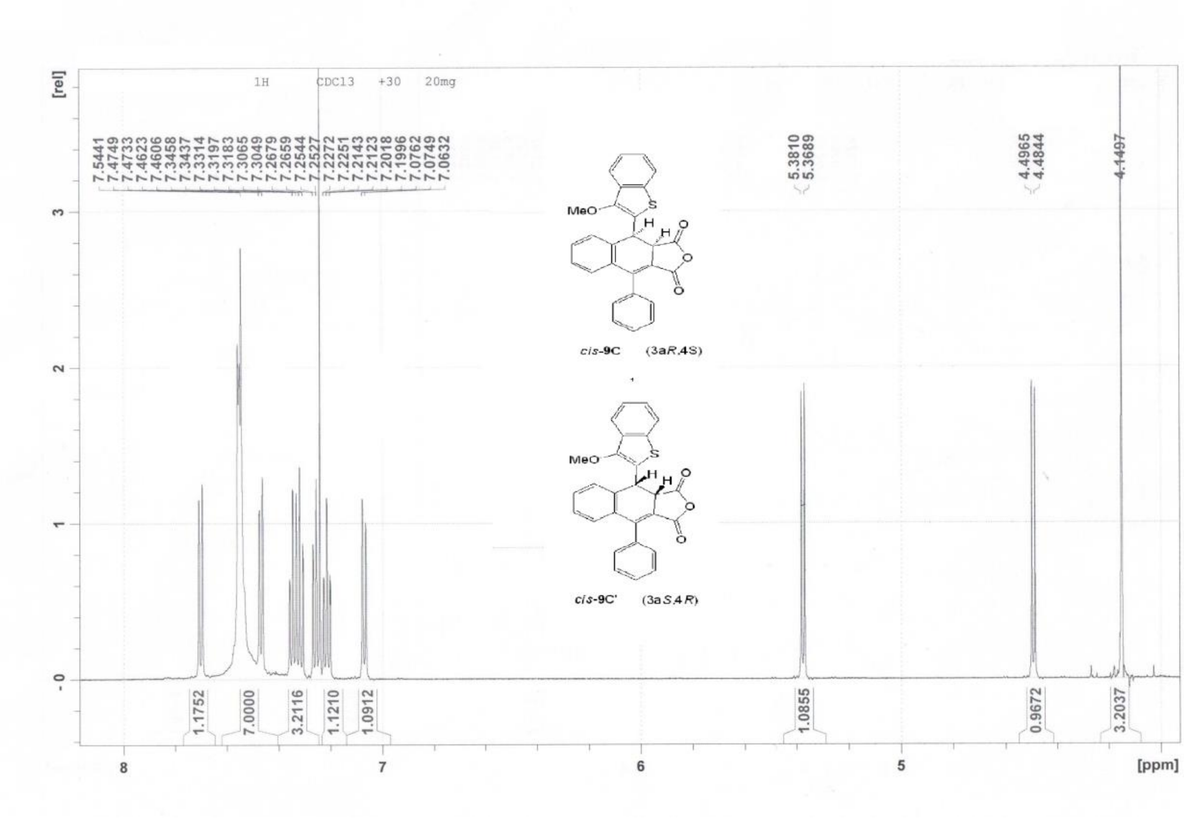

Figure S4: The ^{1}H NMR spectrum of **7*E*** in $CDCl_3$.

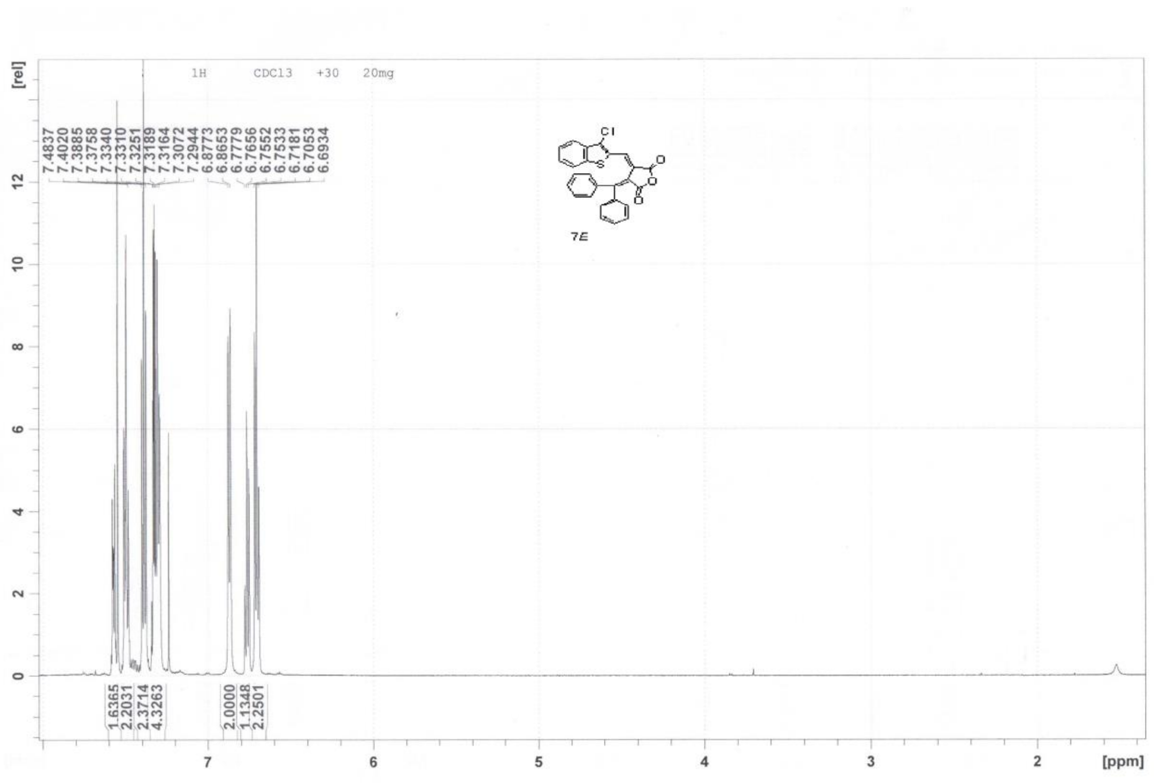

Figure S5: The ^{1}H NMR spectrum of **10**C in $CDCl_3$.

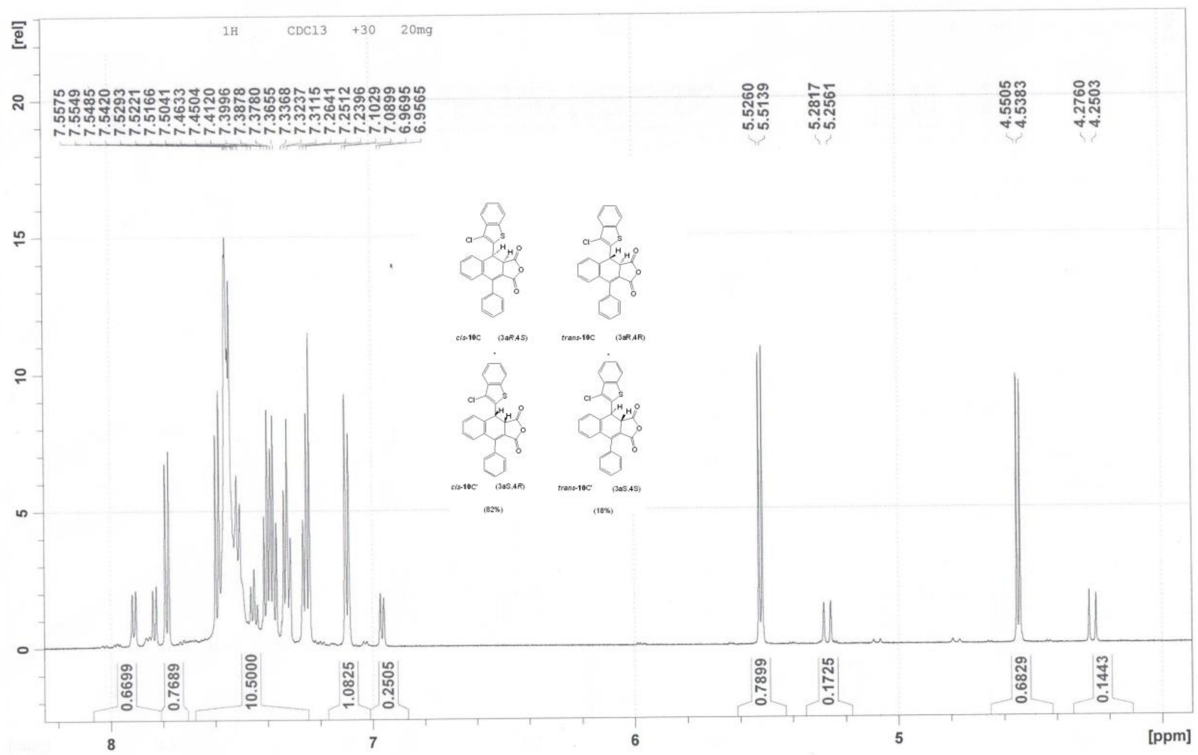

Figure S6: The ^{1}H NMR spectrum of **4***Z* in $CDCl_3$.

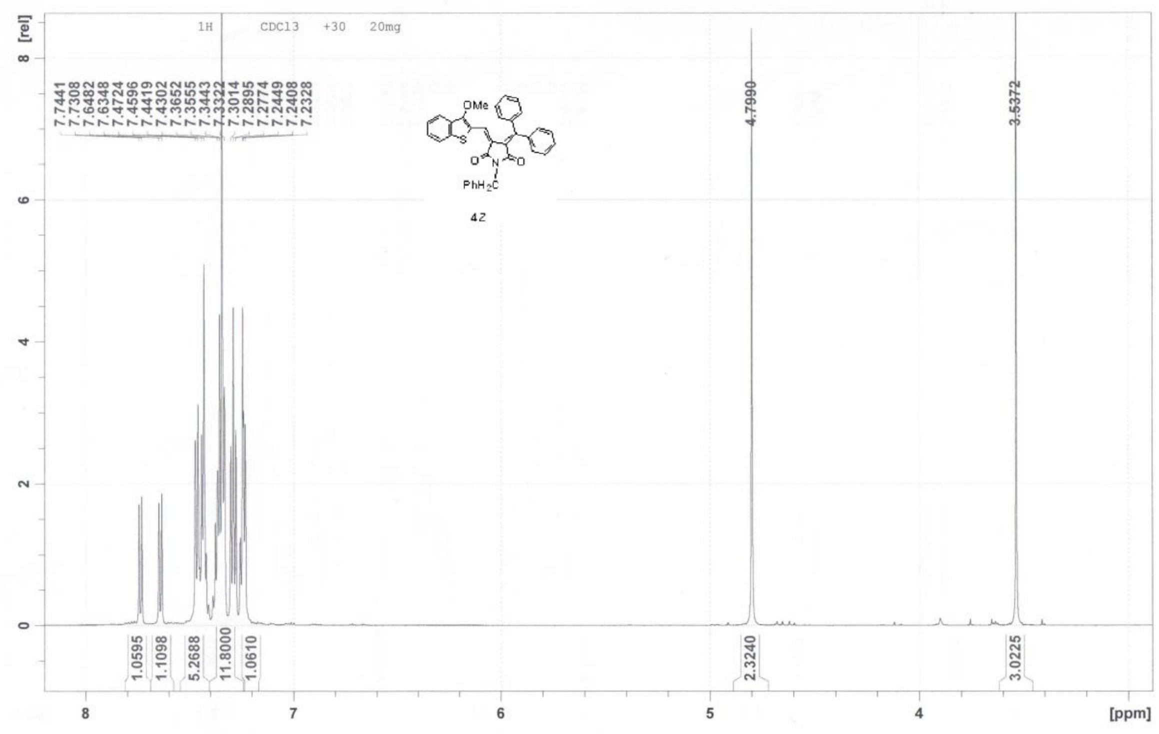

Figure S7: The ^{1}H NMR spectrum of **11**C in $CDCl_3$.

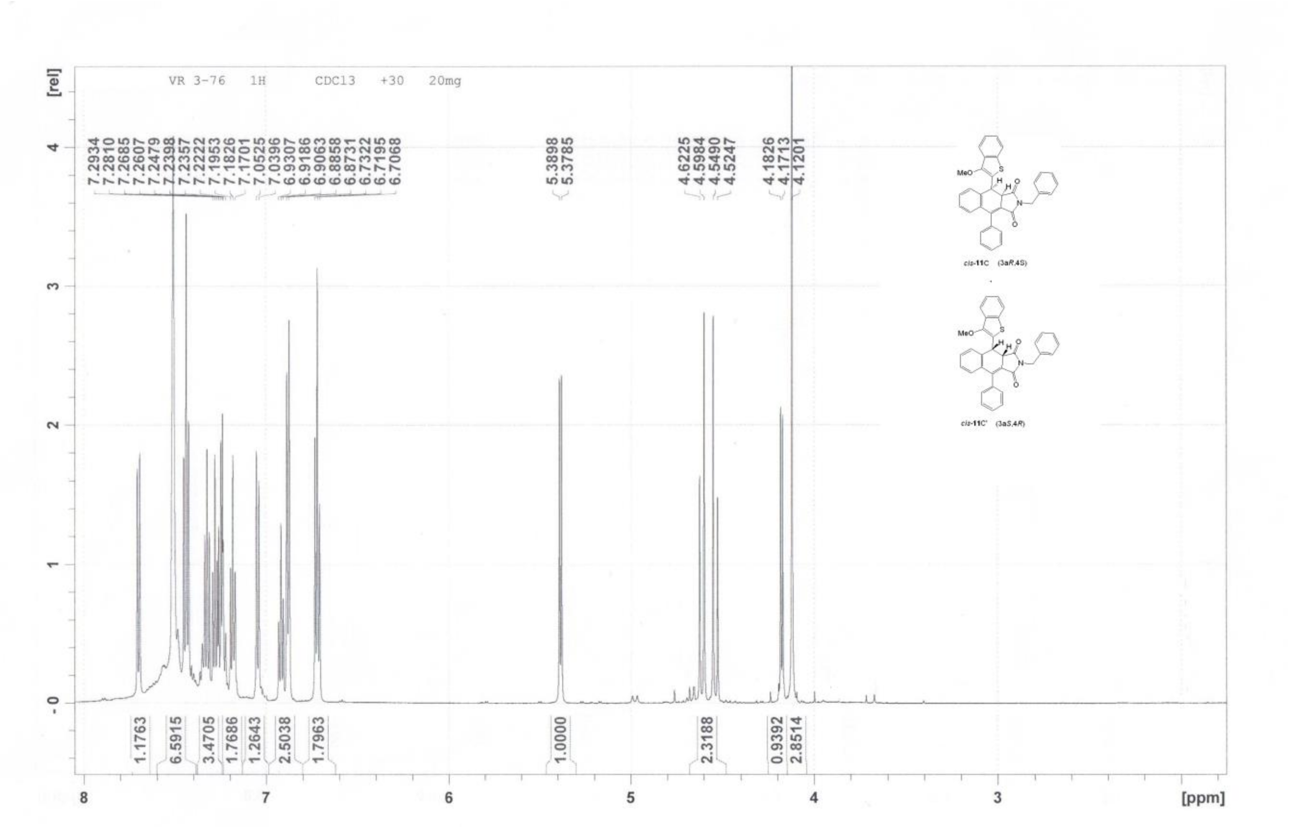

Figure S8: The ^{1}H NMR spectrum of **8*E*** in $CDCl_3$.

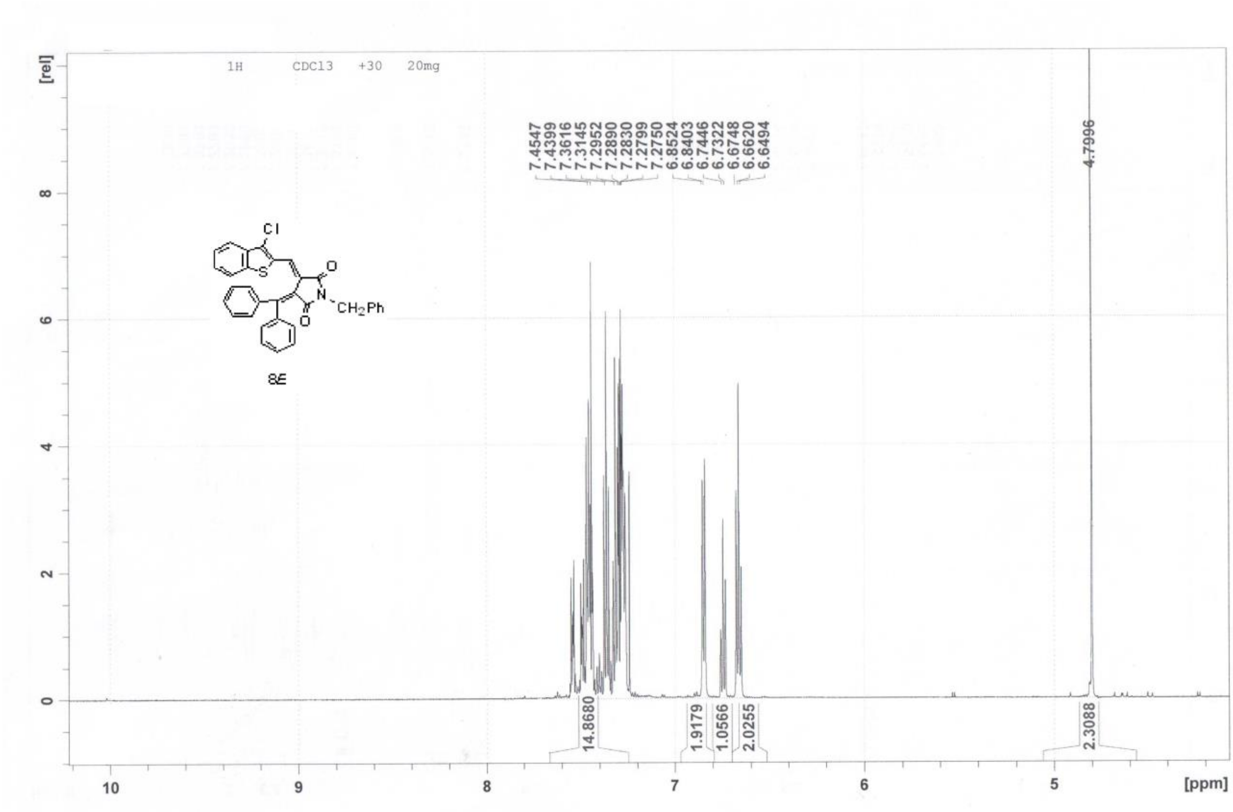

Figure S9: The ^{1}H NMR spectrum of **12**C in $CDCl_3$.

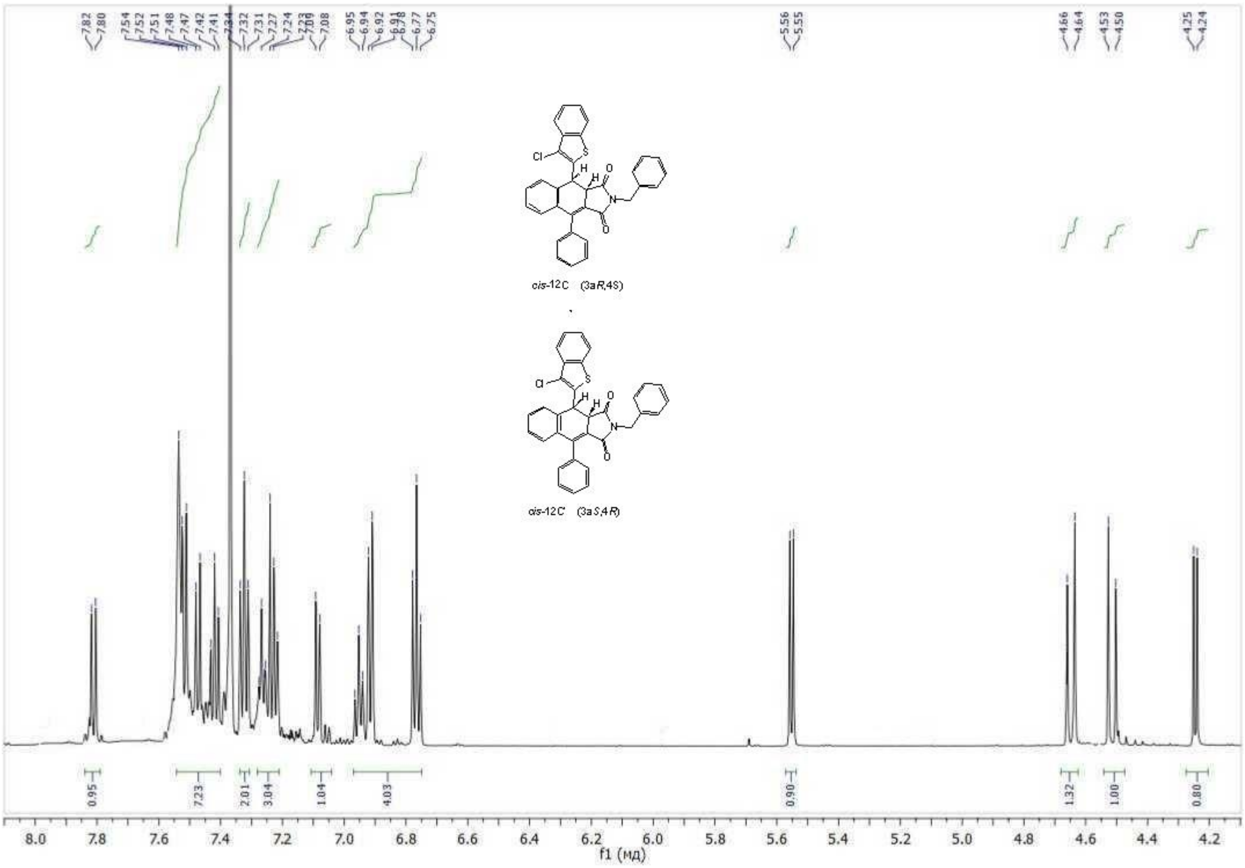

Figure S10: The ^{13}C NMR spectrum of **3*E*** in $CDCl_3$.

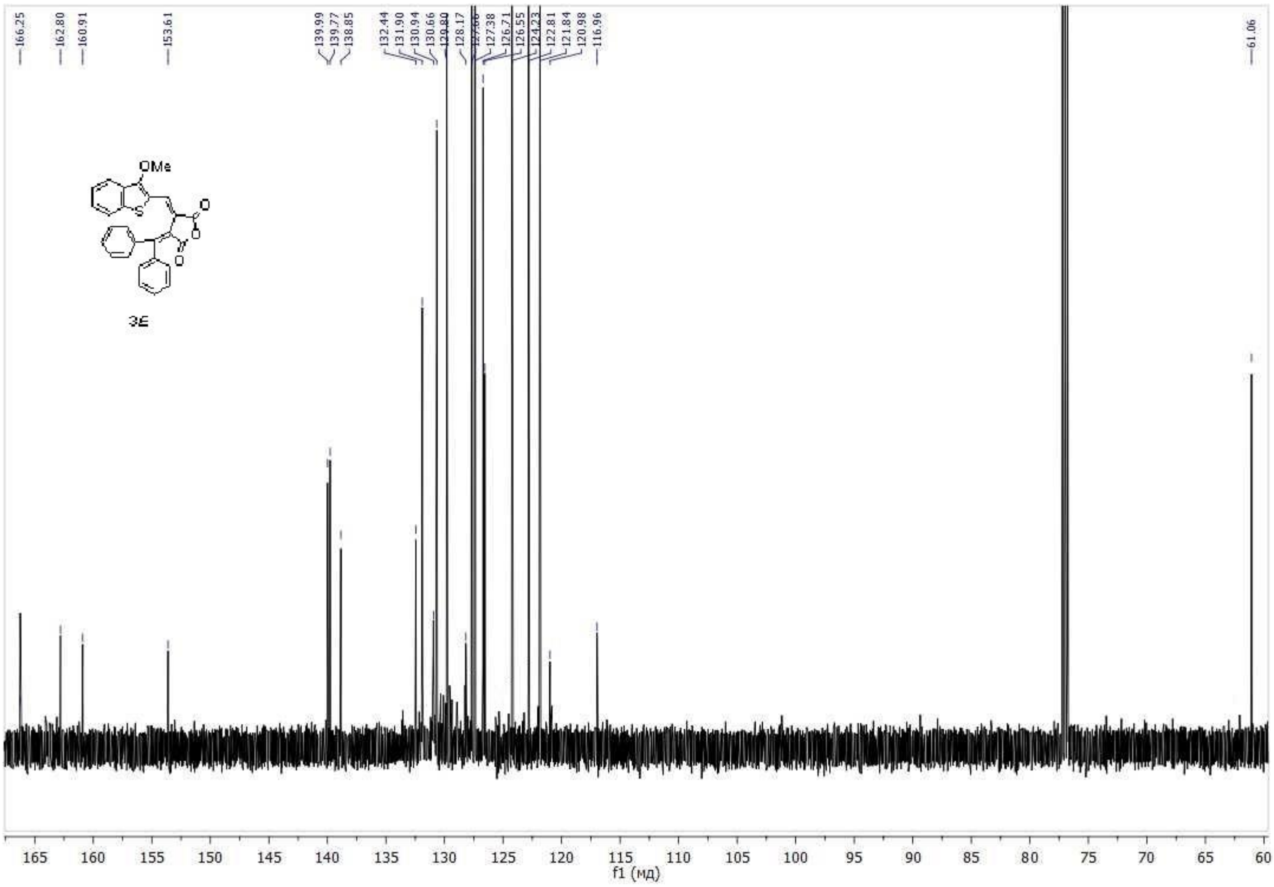

Figure S11: The ^{13}C NMR spectrum of **3***Z* in $CDCl_3$.

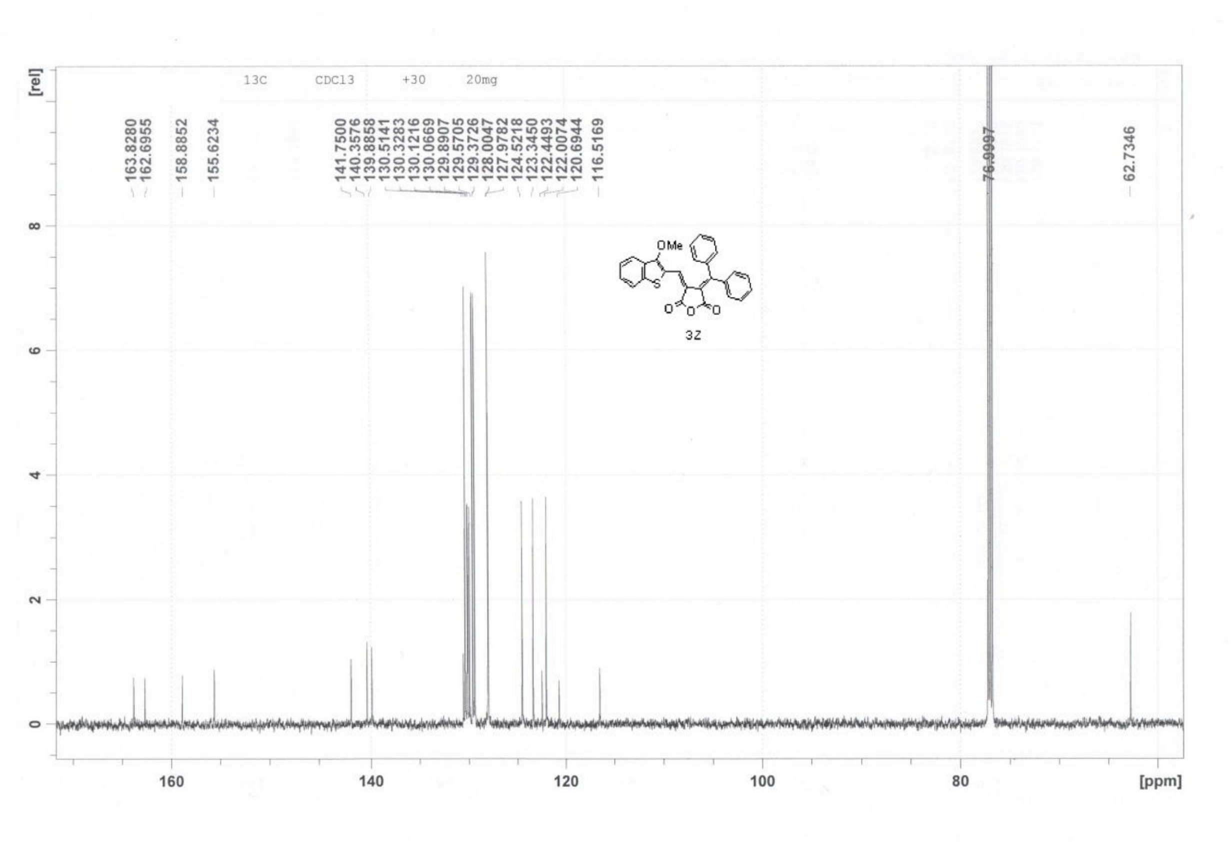

Figure S12: The ^{13}C NMR spectrum of **9**C in $CDCl_3$.

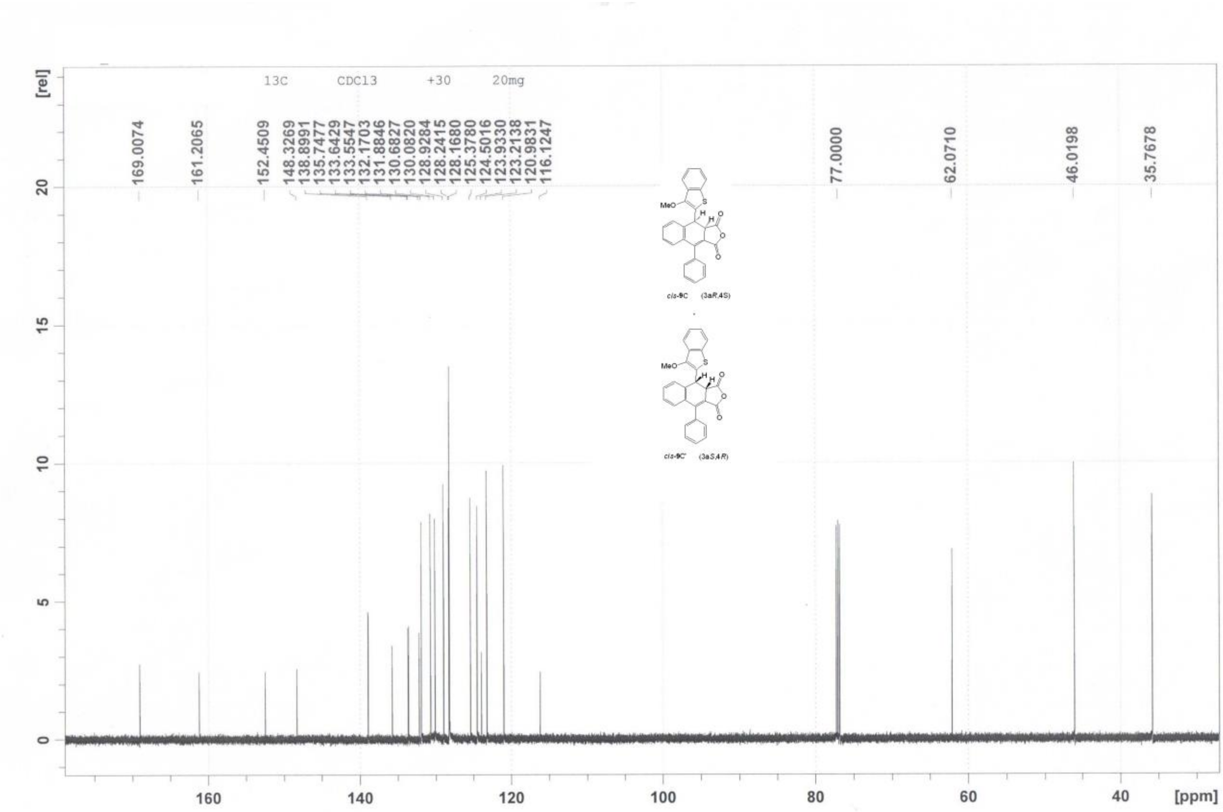

Figure S13: The ^{13}C NMR spectrum of **7*E*** in $CDCl_3$.

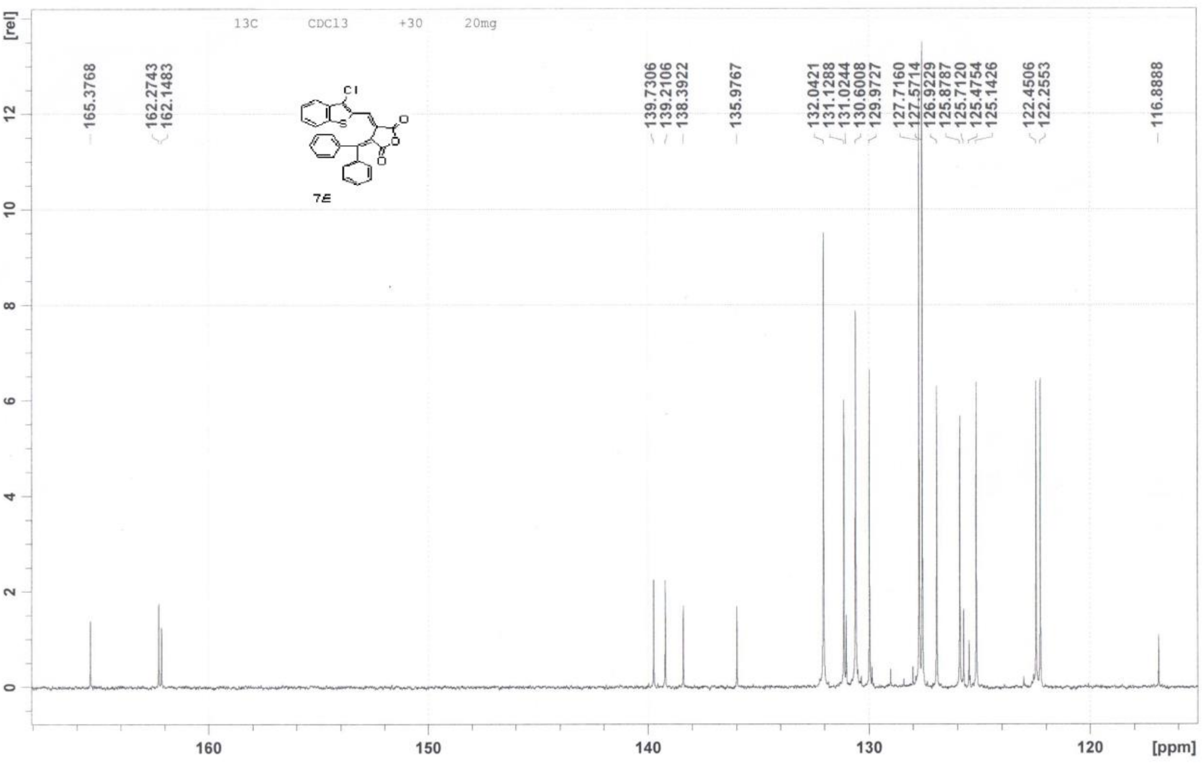

Figure S14: The ^{13}C NMR spectrum of **10**C in $CDCl_3$.

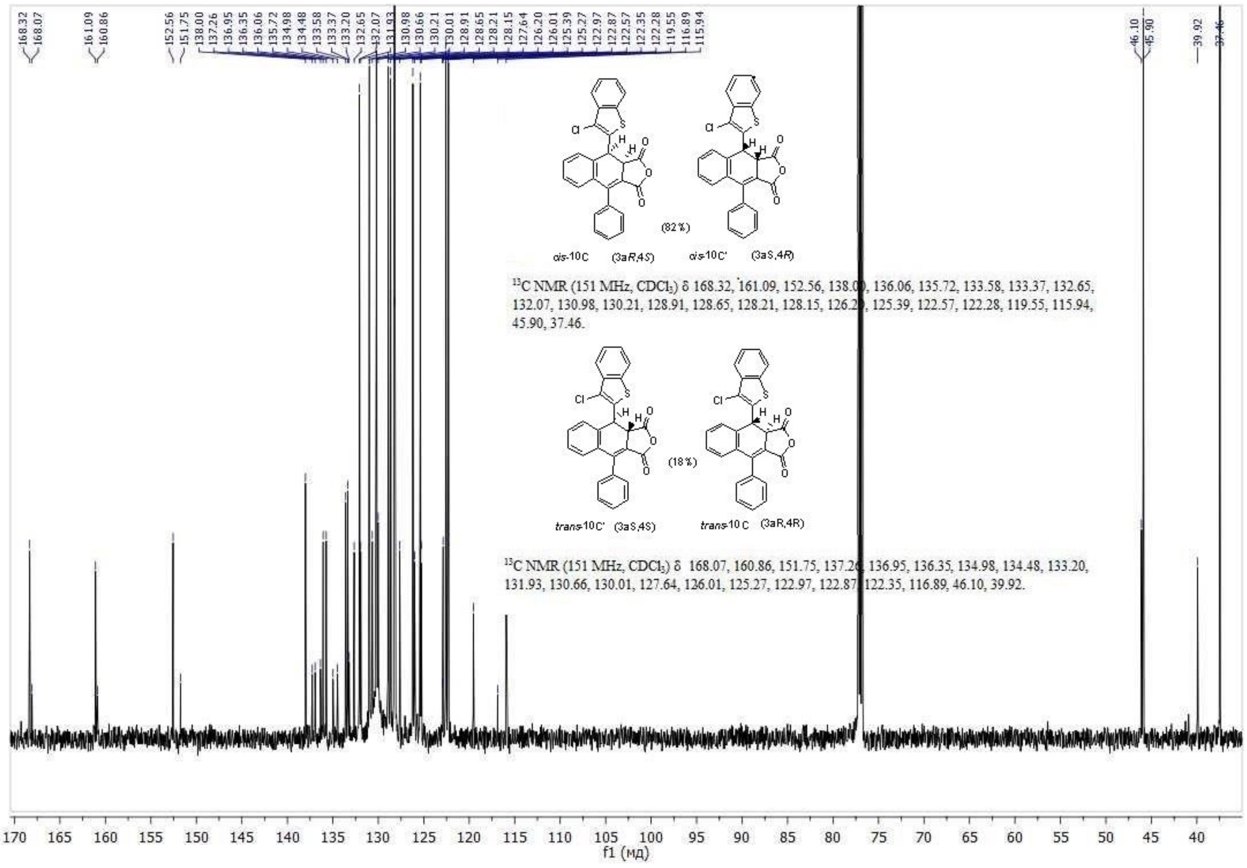

Figure S15: The ^{13}C NMR spectrum of **4***Z* in $CDCl_3$.

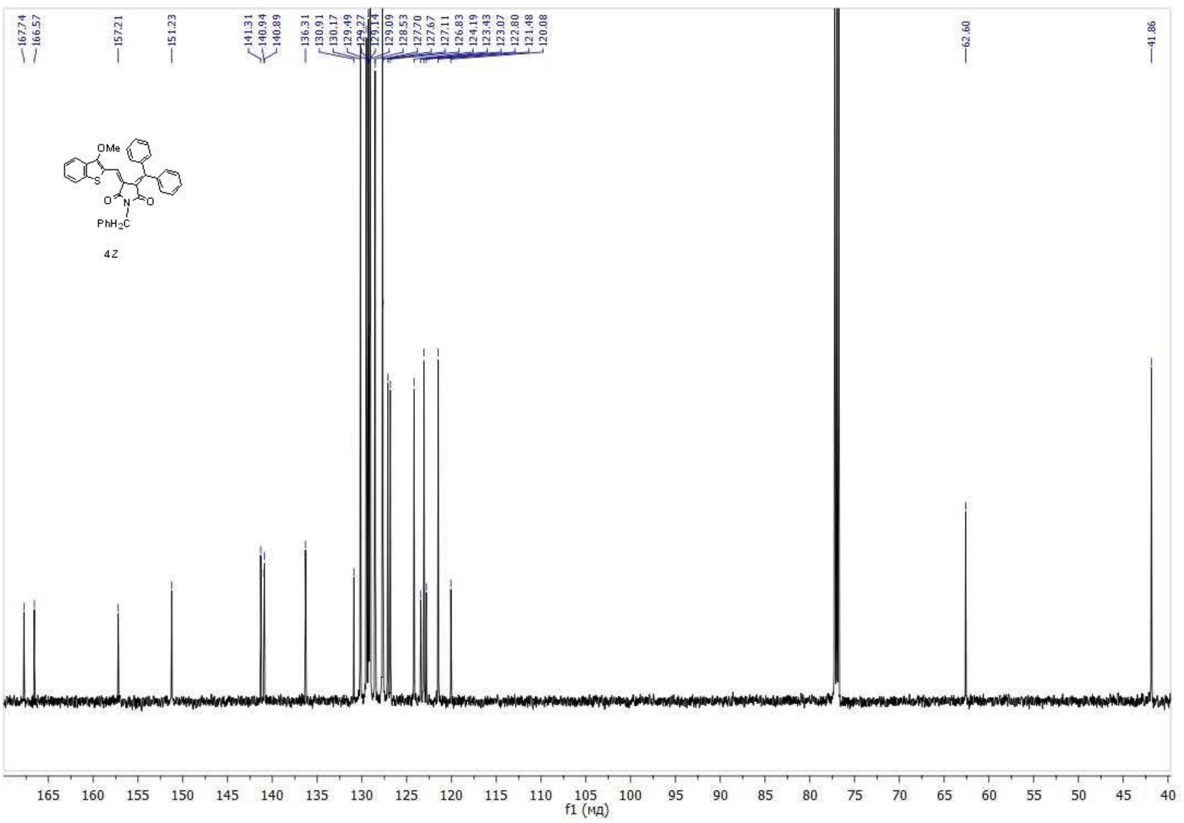

Figure S16: The ^{13}C NMR spectrum of **11**C in $CDCl_3$.

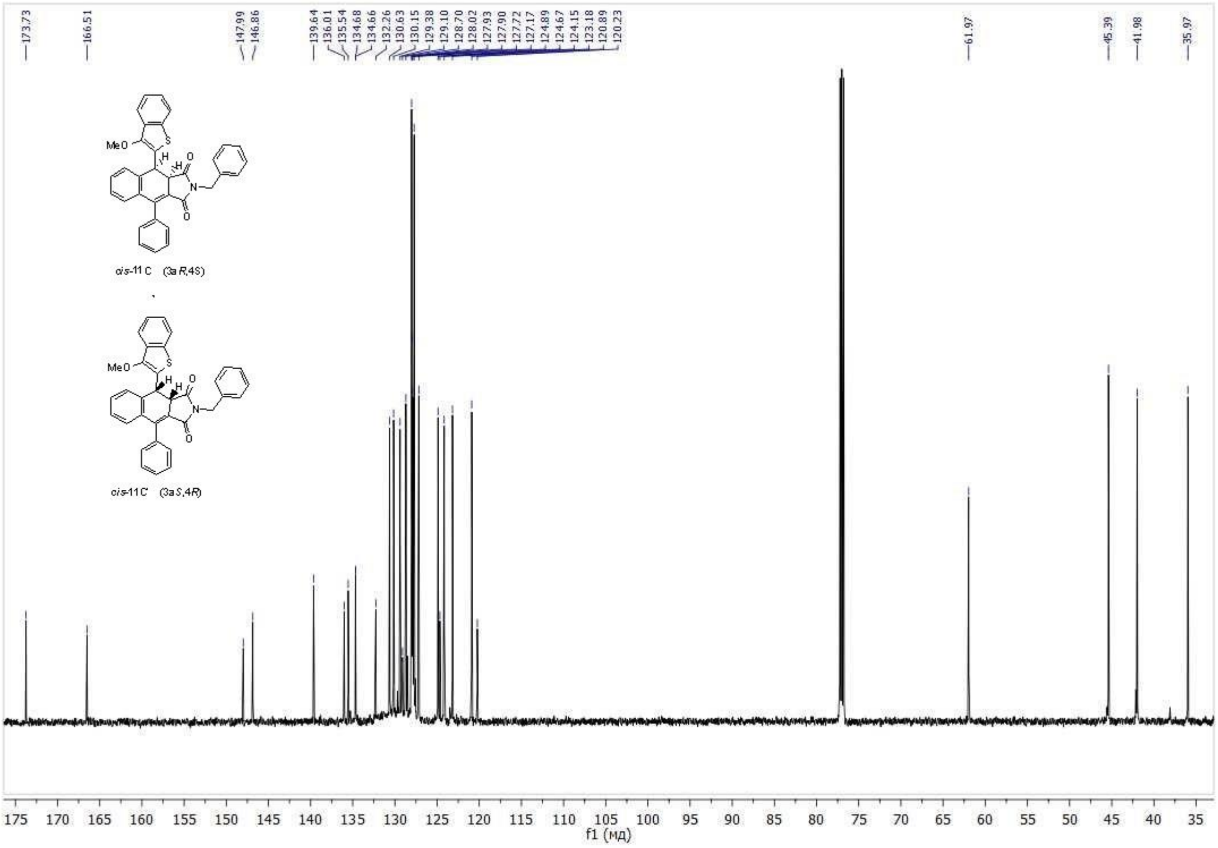

Figure S17: The ^{13}C NMR spectrum of **8***E* in $CDCl_3$.

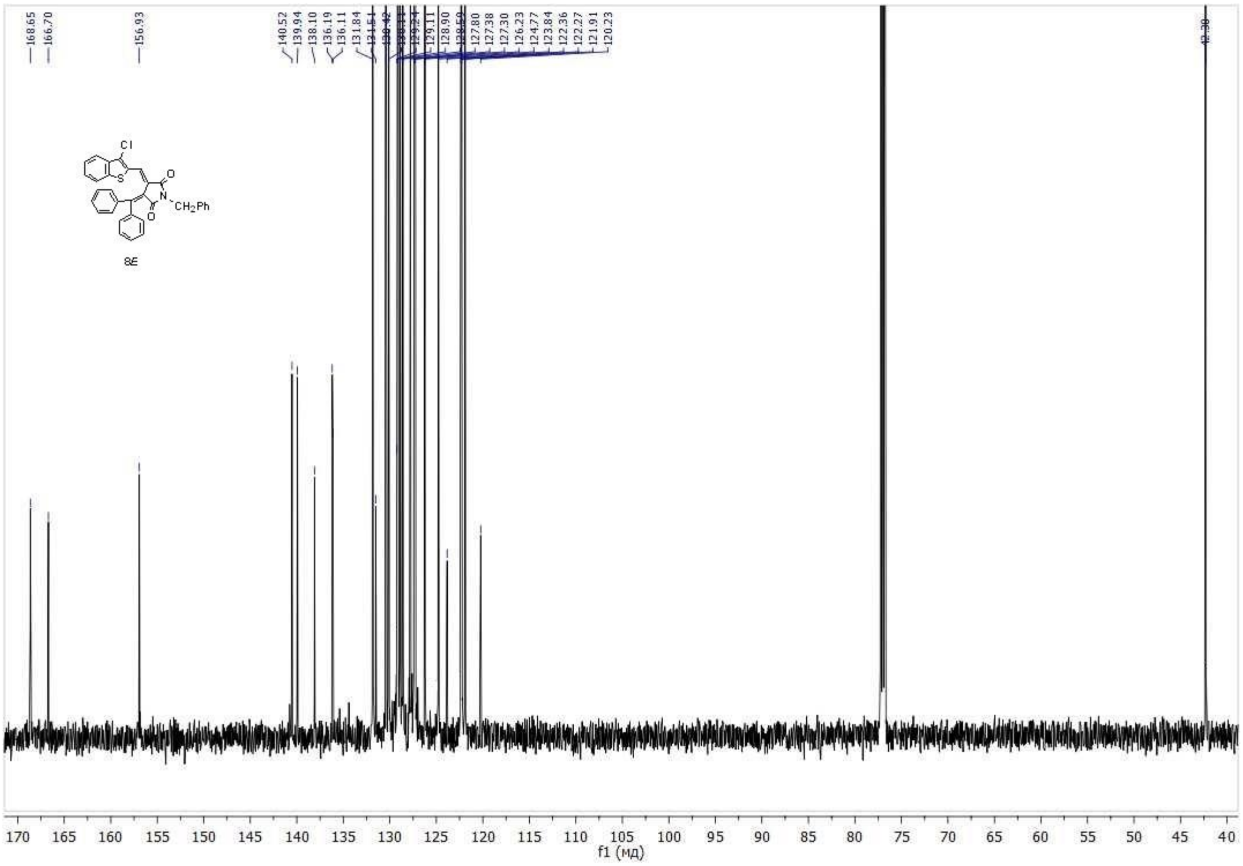

Figure S18: The ^{13}C NMR spectrum of **12**C in $CDCl_3$.

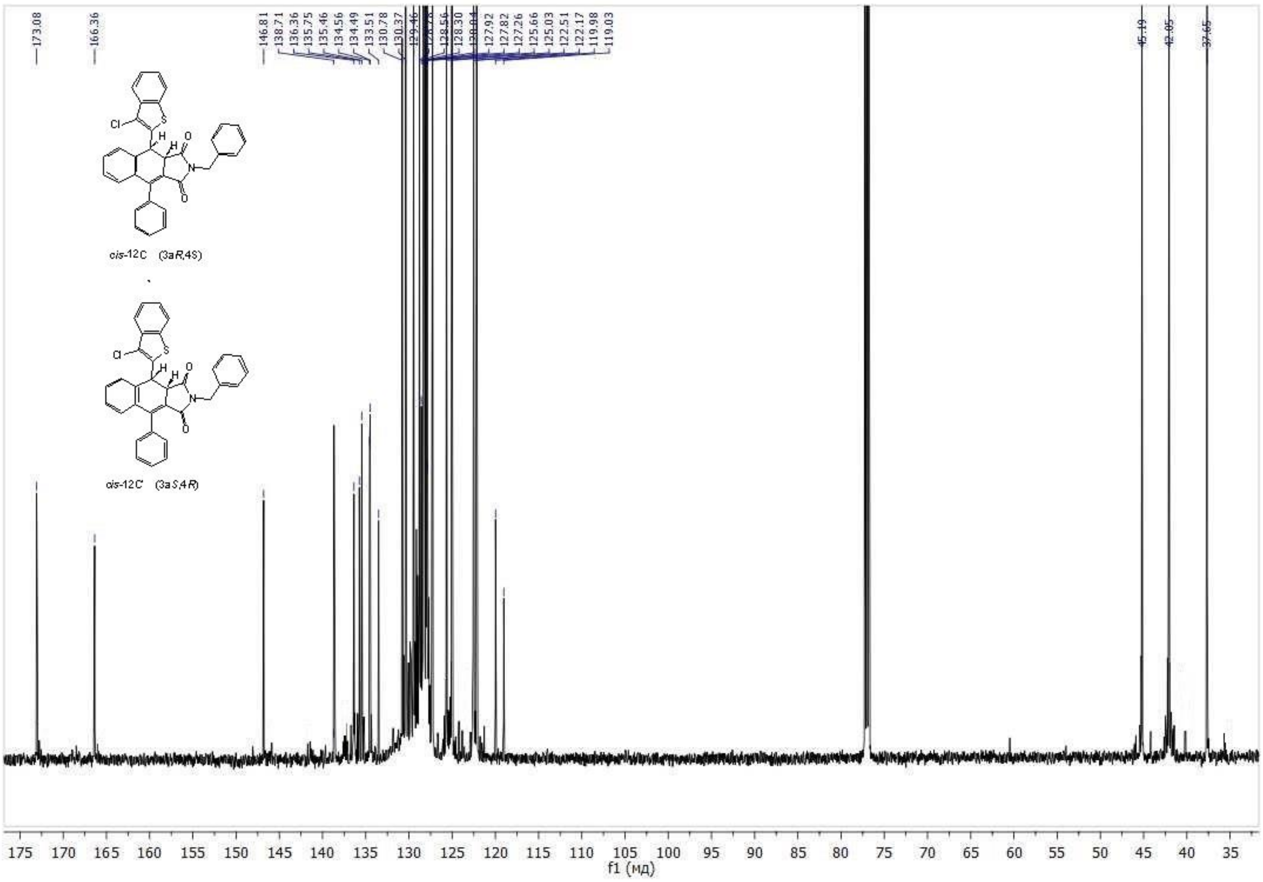

Figure S19: The IR spectra of **3***E* in nujol.

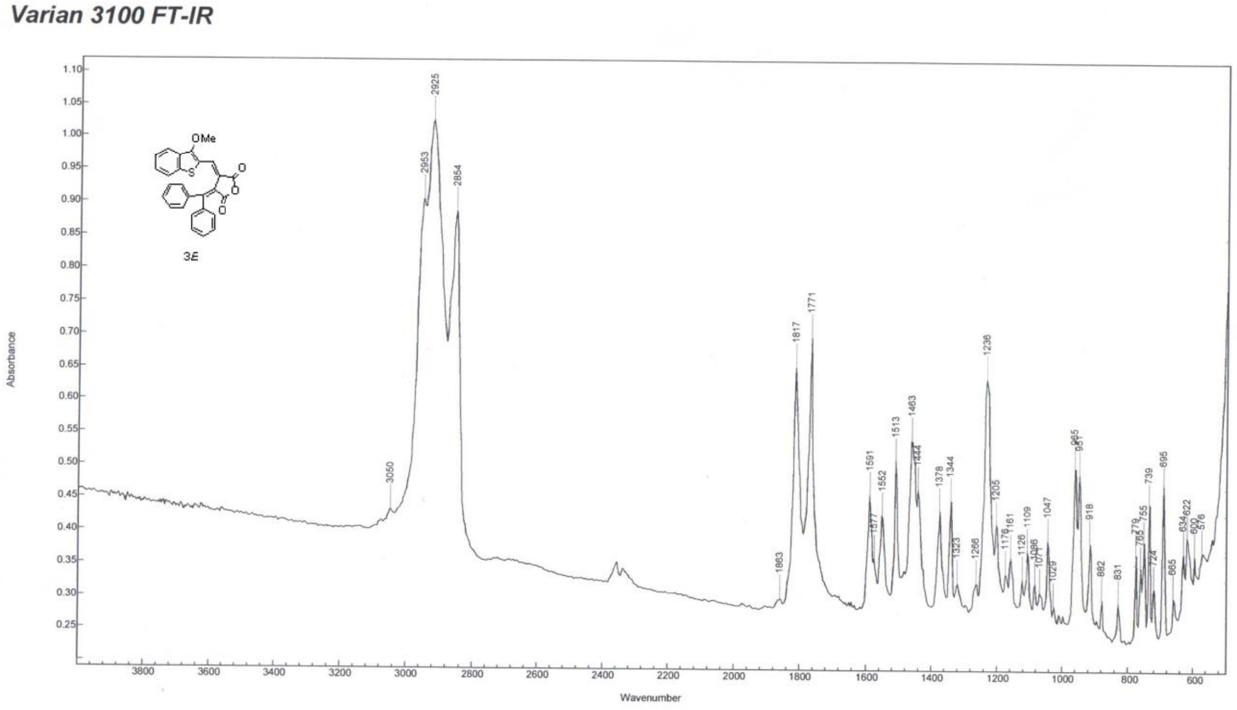

Figure S20: The IR spectra of **3***Z* in nujol.

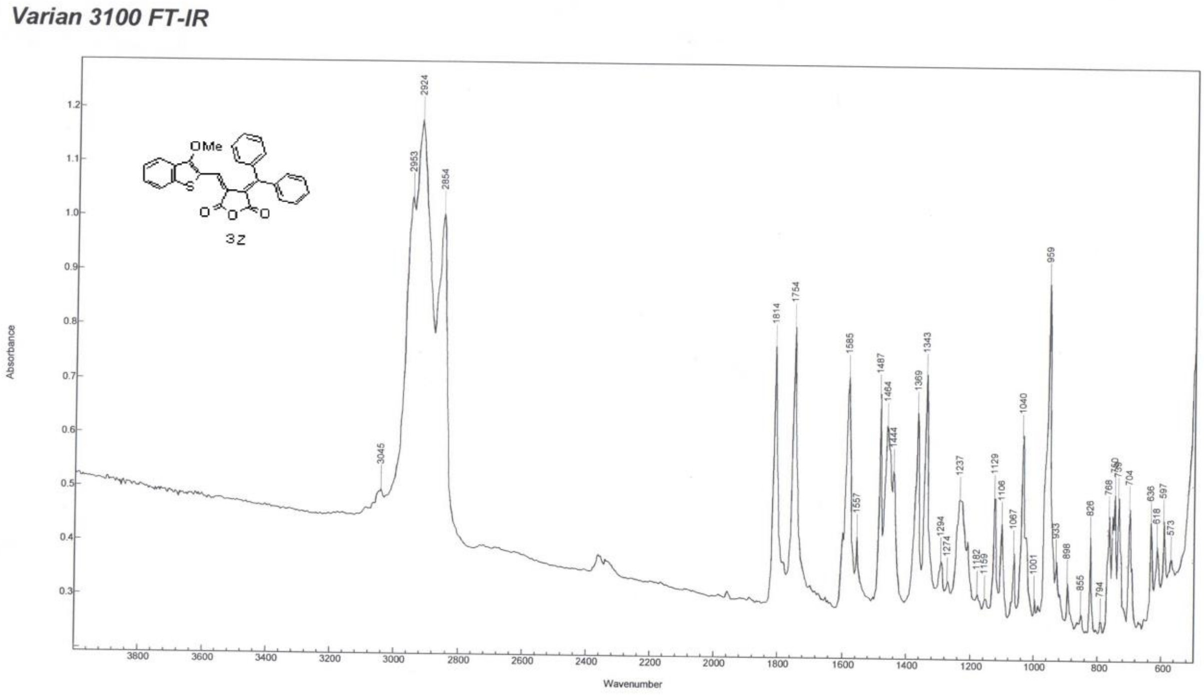

Figure S21: The IR spectra of **9**C in nujol.

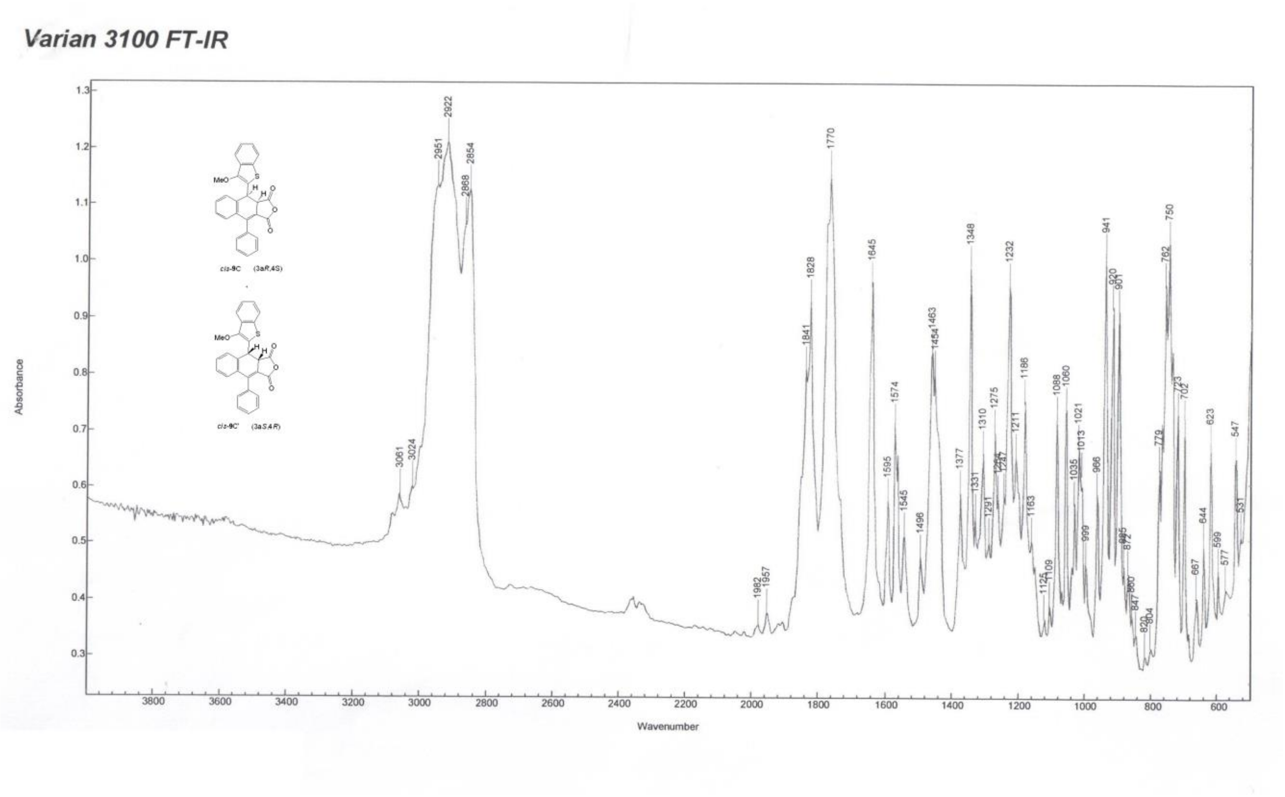

Figure S22: The IR spectra of **7***E* in nujol.

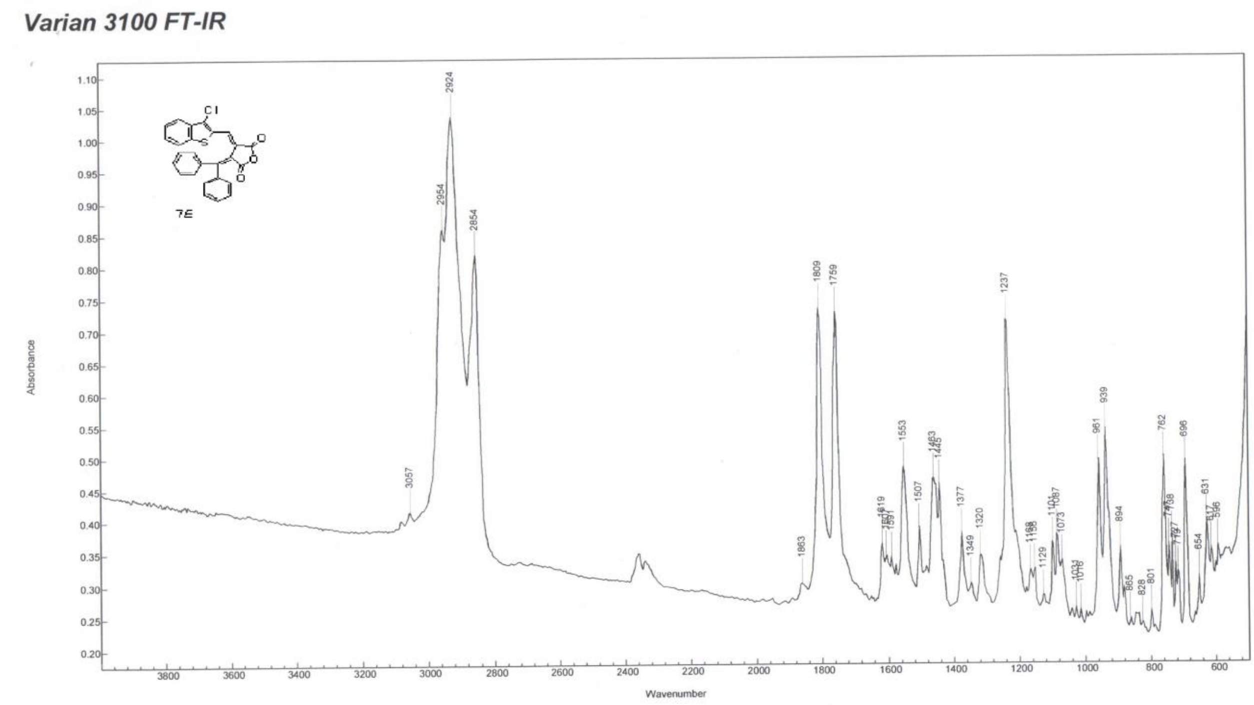

Figure S23: The IR spectra of **10**C in nujol.

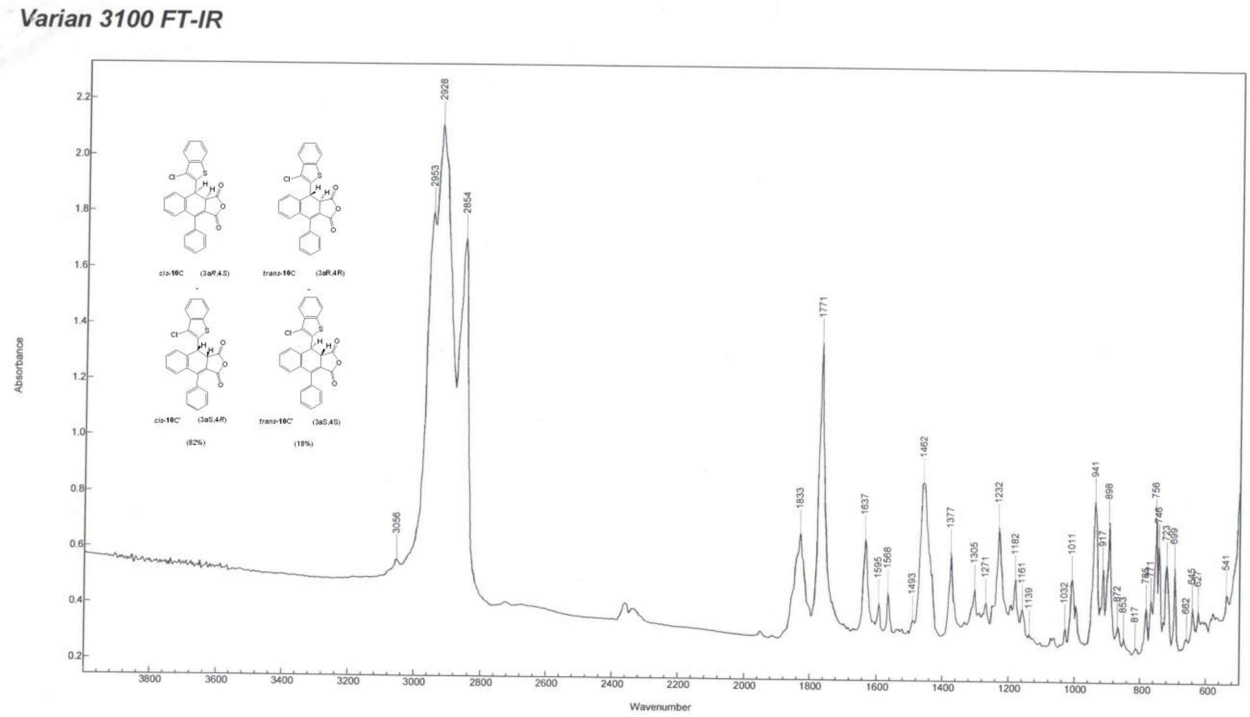

Figure S24: The IR spectra of **4***Z* in nujol.

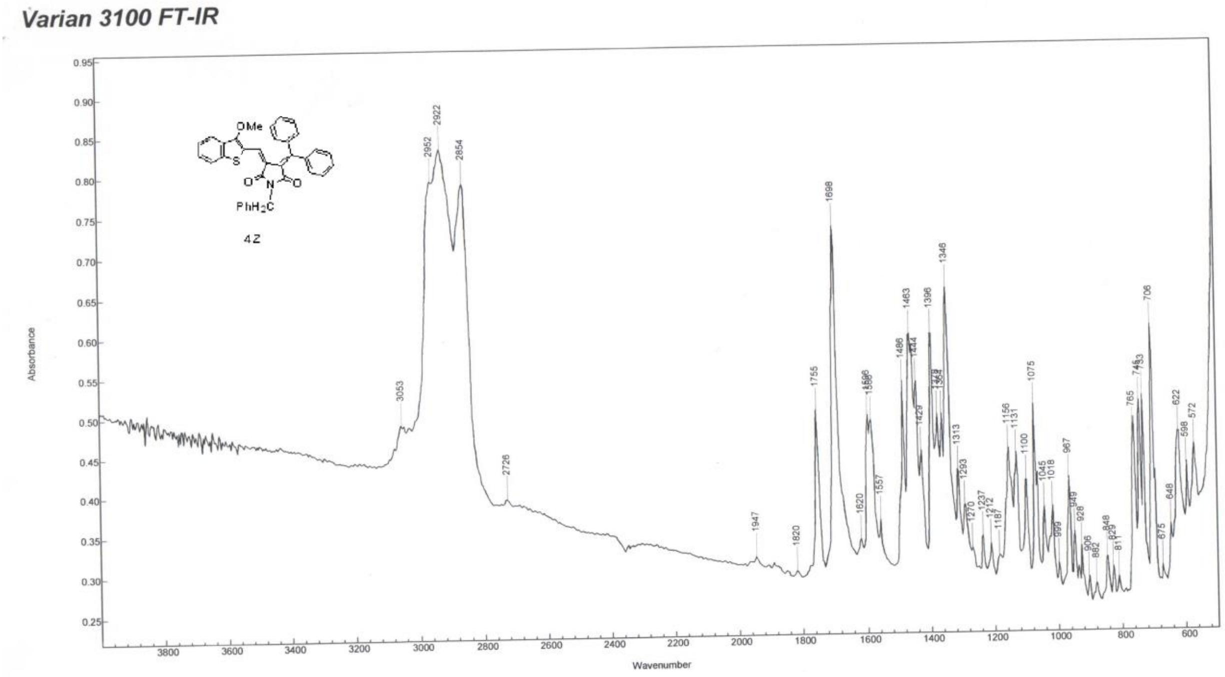

Figure S25: The IR spectra of **11**C in nujol.

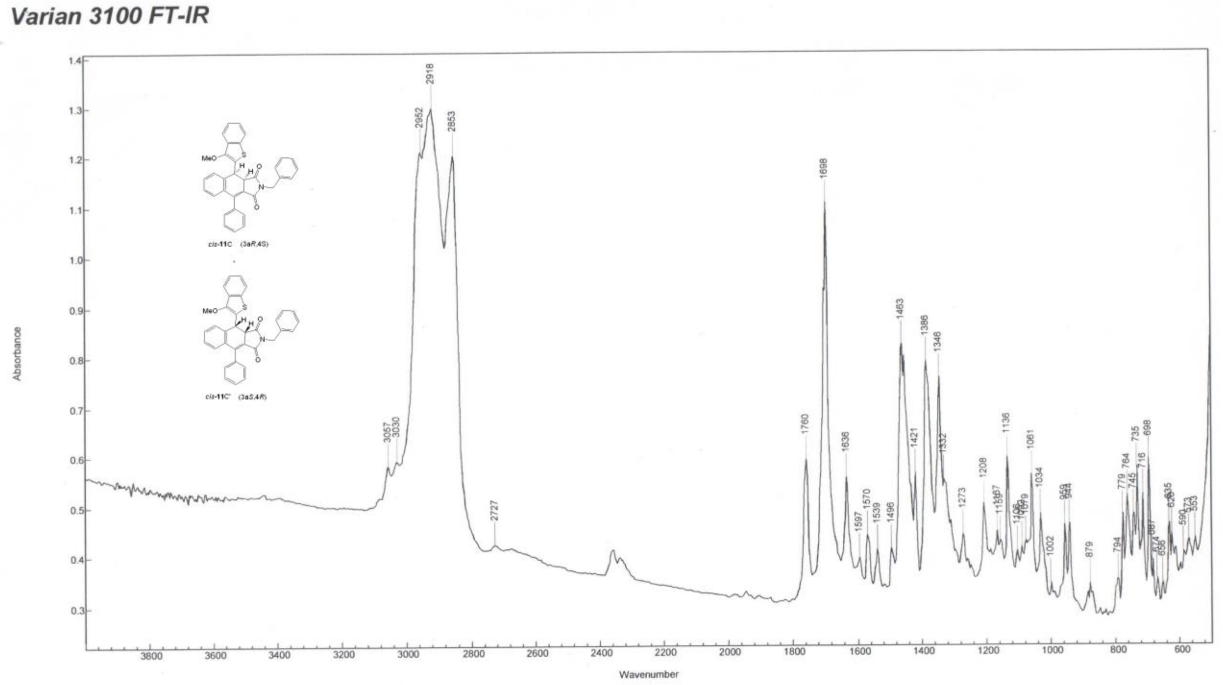

Figure S26: The IR spectra of **8***E* in nujol.

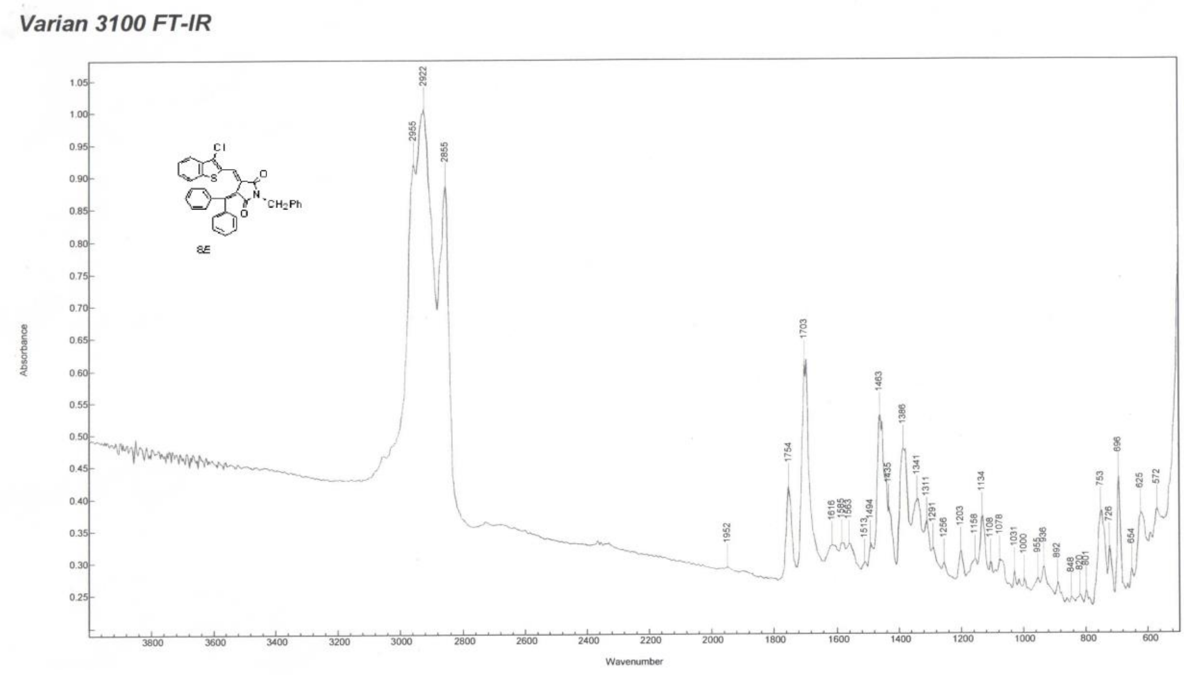

Figure S27: The IR spectra of **12**C in nujol.

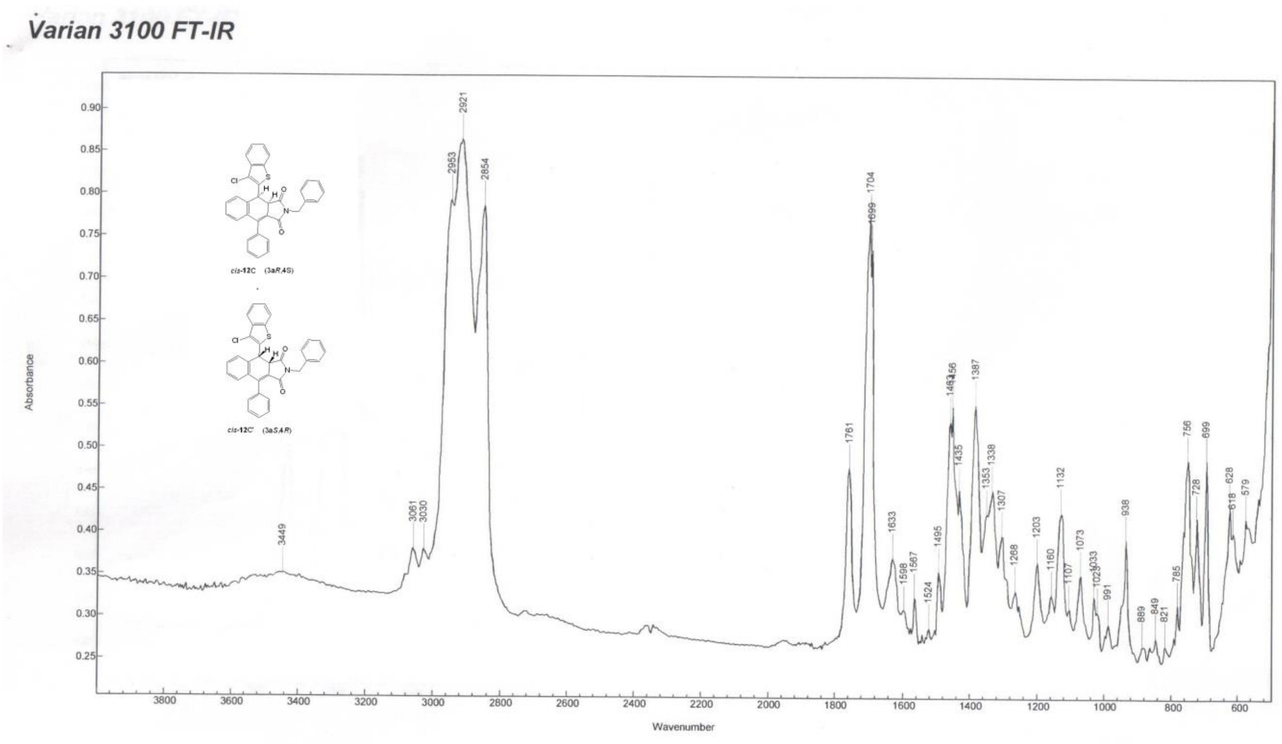

Supplement: File 2 — 1H, 13C NMR and IR spectra of all novel compounds. [file Beilstein_J_Org_Chem-16-1820-s002.pdf]
