# Supplementary material for: Synthesis and highly efficient light-induced rearrangements of diphenylmethylene(2-benzo[b]thienyl)fulgides and fulgimides
Source: Beilstein J Org Chem. 2020 Jul 22;16:1820–9. doi: 10.3762/bjoc.16.149 (PMC7385394; doi:10.3762/bjoc.16.149)
Supplement: File 3 — Experimental procedures and characterization data for fulgides 3E, 3Z, 7E, fulgimides 4Z, 4E, 8E and photo products 9C, 10C, 11C and 12C. [file Beilstein_J_Org_Chem-16-1820-s003.pdf]

BEILSTEIN JOURNAL OF ORGANIC CHEMISTRY

Supporting Information

for

Synthesis and highly efficient light-induced rearrangements of diphenylmethylene(2-benzo[*b*]thienyl)fulgides and fulgimides

Vladimir P. Rybalkin, Sofiya Yu. Zmeeva, Lidiya L. Popova, Valerii V. Tkachev, Andrey N. Utenyshev, Olga Yu. Karlutova, Alexander D. Dubonosov, Vladimir A. Bren, Sergey M. Aldoshin and Vladimir I. Minkin

Beilstein J. Org. Chem. **2020,** *16,* 1820–1829. doi:10.3762/bjoc.16.149

Experimental procedures and characterization data for fulgides 3*E*, 3*Z*, 7*E*, fulgimides 4*Z*, 4*E*, 8*E* and photo products 9C, 10C, 11C and 12C

License and Terms: This is a supporting information file under the terms of the Creative Commons Attribution License (http://creativecommons.org/licenses/by/4.0). Please note that the reuse, redistribution and reproduction in particular requires that the authors and source are credited.
The license is subject to the *Beilstein Journal of Organic Chemistry* terms and conditions: (https://www.beilstein-journals.org/bjoc)

Contents

1. Experimental part……………………………………………………………………3

2. References…………………………………………………………………………9

Synthesis

(*E*)-3-(Diphenylmethylene)-4-((3-methoxybenzo[*b*]thiophen-2-yl-)-methylene)-dihydrofuran-2,5-dione (**3***E*)

Synthesized in a similar manner as described in [1S]. A solution of 3-methoxybenzo[*b*]thiophene-2-carbaldehyde [2S] (10 mmol, 1.9 g) and dimethyl 2-(diphenymethylene)succinate [3S] (10 mmol, 3.1 g) in 20 mL of THF was added dropwise with stirring to a suspension of NaH (12.5 mmol, 0.30 g, 60% dispersion in paraffin) in 10 mL of THF at room temperature. The reaction was initiated with the help of 1-2 drops of methanol. The reaction mixture was left for 1 h after hydrogen stopped evolving. THF was removed on a rotary evaporator. The residue was dissolved in 800 mL of water. The solution was filtered, acidified with 10% aqueous HCl up to pH = 1–2 and extracted with diethyl ether (3 × 15 mL). The diethyl ether was removed on a rotary evaporator. The obtained oil **1** was dissolved in 50 mL of a 10% KOH in methanol and refluxed in a water bath for 8 h. The yellow precipitate was filtered off, rinsed with methanol (5 mL) and dried. Then it was dissolved in 600 mL of water and acidified with 10% aqueous HCl up to pH = 1–2. The colorless precipitate of fulgenic acid **2** was filtered off, rinsed with water (5 mL) and dried. Acid **2** was dissolved in 10 mL of $(EtCO)_2O$ and the reaction mixture was left for 2 h. The precipitate of fulgide **3***E* was filtered off and dried. Yield 3.96 g (90%), red solid, mp 203-204 ^oC. IR ν_{max} (cm^{-1}): 1817, 1771 (C=O). ^1H NMR (600 MHz, $CDCl_3$) δ (ppm): 3.77 (s, 3H, OMe), 6.65-6.72 (m, 3H, arom. H), 6.86-6.89 (m, 2H, arom. H), 7.20-7.53 (m, 9H, arom. H), 7.58 (s, 1H, CH). ^{13}C NMR (600 MHz, $CDCl_3$) δ (ppm): 166.24, 162.80, 160.91, 153.61, 139.99, 139.77, 138.85, 132.44, 131.90, 130.95, 130.66, 129.80, 128.17, 127.66, 127.38, 126.71, 126.55, 124.23, 122.81, 122.79, 121.84, 116.96, 61.28. Anal. Calcd (%) for $C_{27}H_{18}O_4S$: C, 73.96; H, 4.14. Found: C, 73.83; H, 4.25.

(*Z*)-*3*-(Diphenylmethylene)-4-((3-methoxybenzo[*b*]thiophen-2-yl)-methylene)-dihydrofuran-2,5-dione (**3***Z*)

Fulgide **3***E* (0.6 mmol, 0.25 g) was dissolved at heating in 4 mL of benzene in a quartz vessel (50 mL) and irradiated by a Sweko IP65 LED emitter. The solution slowly lightened, evaporated and a light orange precipitate began to form. After the almost complete evaporation, the precipitate was filtered off, rinsed with methanol (5 mL) and dried. Yield 0.21g (84%), light orange solid, mp 244-245 ^oC. IR ν_{max} (cm^{-1}): 1814, 1754 (C=O). ^1H NMR (600 MHz, $CDCl_3$) δ (ppm): 3.61 (s, 3H, OMe), 7.20-7.50 (m, 13H, CH + arom. H), 7.69 (d, 1H, arom. H, *J* = 8.1 Hz); 7.75 (d, 1H, arom. H, *J* = 8.1 Hz). ^{13}C NMR (600 MHz, $CDCl_3$) δ (ppm): 163.83, 162.70, 158.89, 155.62, 141.75, 140.36, 139.89, 130.51, 130.33, 130.12, 130.07, 129.89, 129.57, 129.37, 128.00, 127.98, 124.52, 123.34, 122.45, 122.01, 120.69, 116.52, 62.73. Anal. Calcd (%) for $C_{27}H_{18}O_4S$: C, 73.96; H, 4.14. Found: C, 73.81; H, 4.27.

(*E*)-3-((3-Chlorobenzo[*b*]thiophen-2-yl-)-methylene)-4-diphenylmethylene)-dihydrofuran-2,5-dione (**7***E*)

Synthesized in a similar manner as described in [1S]. A solution of 3-chlorobenzo[*b*]thiophene-2-carbaldehyde [2S] (10 mmol, 1.96 g) and dimethyl 2-(diphenymethylene)succinate [3S] (10 mmol, 3.1 g) in 15 mL of toluene was added dropwise with stirring to a suspension of NaH (15.0 mmol, 0.36 g, 60% dispersion in paraffin) in 5 mL of toluene at room temperature. The reaction was initiated with the help of 1–2 drops of methanol. The reaction mixture was stirred for 2 h and poured into ice/H_2O (300 mL). The organic layer was separated. The water layer was acidified with 10% aqueous HCl up to to pH = 1–2 and extracted with diethyl ether (3 × 45 mL). The diethyl ether was removed on a rotary evaporator. The residue (mono ethyl ester **5**) was dissolved in 30 mL of a 10% KOH in methanol and refluxed in a water bath for 8 h. The yellow precipitate of the potassium salt of fulgenic acid **6** was filtered off, rinsed with

methanol (5 mL) and dissolved in 3 mL of $(EtCO)_2O$ and the reaction mixture was heated at reflux for 1 min. The precipitate of fulgide **3***E* was filtered off, rinsed with methanol (10 mL) and recrystallized from toluene. Yield 2.30 g (52%), red solid, mp 207-209 ^oC. IR ν_{max} (cm^{-1}): 1809, 1759 (C=O). ^1H NMR (600 MHz, $CDCl_3$) δ (ppm): 6.69-6.78 (m, 3H, arom. H), 6.87 (d, 2H, arom. H, J = 7.2 Hz) 7.29-7.51 (m, 8H, arom. H), 7.55 (s, 1H, CH), 7.56-7.58 (m, 1H, arom. H). ^{13}C NMR (600 MHz, $CDCl_3$) δ (ppm): 165.38, 162.27, 162.15, 139.73, 139.21, 138.39, 135.98, 132.04, 131.13, 131.02, 130.60, 129.97, 127.72, 127.57, 126.92, 125.88, 125.71, 125.47, 125.14, 122.45, 122.25, 116.89. Anal. Calcd (%) for $C_{26}H_{15}ClO_3S$: C, 70.51; H, 3.41. Found: C, 70.37; H, 3.55.

Synthesis of fulgimides **4***Z*, **4***E* and **8***E* (general methodics)

A solution of benzylamine (11 mmol, 0.12 g) in 5 mL of benzene was added dropwise to a solution of fulgide **3***Z*, **3***E* or **7***E* (11 mmol) in 12 mL of benzene. The reaction mixture was stirred for 1 h at room temperature and then $ZnCl_2$ (11 mmol, 0.15 g) was added. Hexamethyldisilazane (16 mmol, 0.33 mL) was added to the reaction mixture heated to 80 ^oC and then the mixture was heated at reflux for 4 h. The reaction mixture was rinsed with 25% aqueous NH_4OH (40 mL), the organic layer was separated. The solvent was removed on a rotary evaporator. The obtained orange oil was purified by column chromatography (sorbent SiO_2, eluent CH_2Cl_2). The CH_2Cl_2 was removed on a rotary evaporator and the obtained orange solid was dried in air. From fulgides **3***Z* and **7***E*, fulgimides **4***Z* and ***8E***, correspondingly, were obtained. Fulgide **3***E* gave rise to a mixture of fulgimides **4***E* (41%) and **4***Z* (18%).

(*Z*)-1-Benzyl-3-(diphenylmethylene)-4-((3-methoxybenzo[*b*]thiophen-2-yl)-methylene)-pyrrolidine-2,5-dione (**4***Z*)

Yield 0.24 g (41%), orange solid, mp 195-197 ^oC (*n*-BuOH). IR ν_{max} (cm^{-1}): 1755, 1698 (C=O). ^1H NMR (600 MHz, $CDCl_3$) δ (ppm): 3.54 (s, 3H, OMe), 4.80 (s, 2H, CH_2), 7.20-

7.50 (m, 18H, CH + arom. H), 7.64 (d, 1H, arom. H, *J* = 8.0 Hz), 7.74 (d, 1H, arom. H, *J* = 7.9 Hz). ^{13}C NMR (600 MHz, $CDCl_3$) δ (ppm): 167.74, 166.57, 157.21, 151.23, 141.31, 140.94, 140.89, 136.31, 130.91, 130.17, 129.49, 129.27, 129.14, 129.09, 128.53, 127.70, 127.67, 127.11, 126.83, 124.19, 123.43, 123.07, 122.80, 121.48, 120.08, 62.60, 41.86. Anal. Calcd (%) for $C_{34}H_{25}NO_3S$: C, 77.40; H, 4.78; N, 2.65. Found: C, 77.31; H, 4.91; N, 2.52.

(*E*)-1-Benzyl-3-(diphenylmethylene)-4-((3-methoxybenzo[*b*]thiophen-2-yl)-methylene)-pyrrolidine-2,5-dione (**4***E*)

Yield 0.10 g (18%), orange solid, mp 143-144 ^{o}C (*n*-BuOH). IR ν_{max} (cm^{-1}): 1750, 1698 (C=O). ^{1}H NMR (600 MHz, $CDCl_3$) δ (ppm): 3.74 (s, 3H, OMe), 4.78 (s, 2H, CH_2), 6.60-6.95 (m, 3H, arom. H), 7.14 (d, 1H, arom. H, *J* = 8.0 Hz), 7.52 (m, 17H, CH + arom. H). Anal. Calcd (%) for $C_{34}H_{25}NO_3S$: C, 77.40; H, 4.78; N, 2.65. Found: C, 77.51; H, 4.69; N, 2.79.

(*E*)-1-Benzyl-3-((3-chlorobenzo[*b*]thiophen-2-yl)-methylene)-4-(diphenylmethylene)-pyrrolidine-2,5-dione (**8***E*)

Yield 0.34 g (18%), orange solid, mp 112-113 ^{o}C (*n*-BuOH). IR ν_{max} (cm^{-1}): 1754, 1703 (C=O). ^{1}H NMR (600 MHz, $CDCl_3$) δ (ppm): 4.80 (s, 2H, CH_2), 6.64-6.86 (m, 5H, arom. H), 7.27-7.46 (m, 15H, CH + arom. H). ^{13}C NMR (600 MHz, $CDCl_3$) δ (ppm): 168.65, 166.70, 156.93, 140.52, 139.94, 138.10, 136.19, 136.11, 131.84, 131.51, 130.42, 130.11, 129.24, 129.11, 128.90, 128.59,127.80, 127.38, 127.30, 126.23, 124.77, 123.84, 122.36, 122.27, 121.91, 120.23, 42.30. Anal. Calcd (%) for $C_{33}H_{22}ClNO_2S$: C, 74.50; H, 4.17. N, 2.63. Found: C, 74.35; H, 4.32; N, 2.49.

(3a*R*,4*S*)-4-(3-Methoxybenzo[*b*]thiophen-2-yl)-9-phenyl-3a,4-dihydronaphtho[2,3-*c*]furan-1,3-dione (*cis*-**9**C) and (3a*S*,4*R*)-4-(3-methoxybenzo[*b*]thiophen-2-yl)-9-phenyl-3a,4-dihydronaphtho[2,3-*c*]furan-1,3-dione (*cis*-**9**C')

Fulgide **3***E* or **3***Z* (0.6 mmol, 0.25 g) was dissolved at heating in 4 mL of benzene in a quartz vessel (50 mL) and irradiated by a Sweko IP65 LED emitter. The solution slowly evaporated and a light orange precipitate of **3***Z* formed. $CHCl_3$ (2 mL) and CH_3CN (2 mL) was added to the reaction mixture and the solution was irradiated until almost complete decoloration and evaporation. Colorless solid was filtered off, rinsed with methanol (5 mL), dried and recrystallized from CH_3CN. Yield 0.21g (84%), colorless solid, mp 142 °C. IR ν_{max} (cm^{-1}): 1841, 1828, 1770 (C=O). ^{1}H NMR (600 MHz, $CDCl_3$) δ (ppm): 4.15 (s, 3H, OMe), 4.49 (d, 1H, CH, *J* = 7.3 Hz), 5.37 (d, 1H, CH, *J* = 7.3 Hz), 7.07 (d, 1H, arom. H, *J* = 7.0 Hz), 7.20-7.36 (m, 4H, arom. H), 7.47 (d, 1H, arom. H, *J* = 6.6 Hz),7.47-7.64 (m, 6H, arom. H), 7.70 (d, 1H, arom. H, *J* = 6.8 Hz). ^{13}C NMR (600 MHz, $CDCl_3$) δ (ppm): 169.01, 161.21, 152.45, 148.33, 138.90, 135.75, 133.64, 133.55, 132.17, 131.89, 130.68, 130.08, 128.93, 128.24, 128.17, 125.38, 124.50, 123.93, 123.21, 120.98, 116.12, 62.07, 46.02, 35.77. Anal. Calcd (%) for $C_{27}H_{18}O_4S$: C, 73.96; H, 4.14. Found: C, 74.15; H, 3.97.

(3a*R*,4*S*)-4-(3-Chlorobenzo[*b*]thiophen-2-yl)-9-phenyl-3a,4-dihydronaphtho[2,1-*c*]furan-1,3-dione (*cis*-**10**C) and (3a*S*,4*R*)-4-(3-chlorobenzo[*b*]thiophen-2-yl)-9-phenyl-3a,4-dihydronaphtho[2,1-*c*]furan-1,3-dione (*cis*-**10**C')

Fulgide **7***E* (0.6 mmol, 0.25 g) was dissolved at heating in 2.5 mL of benzene in a quartz vessel (50 mL) and irradiated by a Sweko IP65 LED emitter. After slow complete dissolution of fulgide, CH_3CN (1 mL) was added to the reaction mixture and the solution was irradiated until almost complete decoloration and evaporation. The colorless solid was filtered off, rinsed with methanol (5 mL), dried and recrystallized from CH_3CN. Yield 0.22 g (86 %), colorless solid, mp 220-223 °C. IR ν_{max} (cm^{-1}): 1833, 1771 (C=O). According to NMR spectra a mixture of isomers *cis*-**10**C (82%) and *trans*-**10**C (18%) was obtained. ^{1}H NMR (600 MHz, $CDCl_3$) δ (ppm): 4.54 (d, 1H, CH, *J* = 7.3 Hz), 5.52 (d, 1H, CH, *J* = 7.3 Hz), 7.8 (d, 1H, arom. H, *J* = 7.0 Hz), 7.23-7.64 (m, 11H, arom. H), 7.78

(d, 1H, arom. H, *J* = 8.4 Hz). ^{13}C NMR (600 MHz, $CDCl_3$) *δ* (ppm): 168.32, 161.09, 152.56, 138.00, 136.06, 135.72, 133.58, 133.37, 132.65, 132.07, 130.98, 130.21, 128.91, 128.65, 128.21, 128.15, 126.20, 125.39, 122.57, 122.28, 119.55, 115.94, 45.90, 37.46. Anal. Calcd (%) for $C_{26}H_{15}ClO_3S$: C, 70.51; H, 3.41. Found: C, 70.34; H, 3.53.

(3a*R*,4*R*)-4-(3-Chlorobenzo[*b*]thiophen-2-yl)-9-phenyl-3a,4-dihydronaphtho[2,3-*c*]-furan-1,3-dione (*trans*-**10**C) and (3a*S*,4*S*)-4-(3-chlorobenzo[*b*]thiophen-2-yl)-9-phenyl-3a,4-dihydronaphtho[2,3-*c*]-furan-1,3-dione (*trans*-**10**C')

Synthesized according to the previous methodics. ^{1}H NMR (600 MHz, $CDCl_3$) *δ* (ppm): 4.26 (d, 1H, CH, *J* = 15.4 Hz), 4.54 (d, 1H, CH, *J* = 15.4 Hz), 6.96 (d, 1H, arom. H, *J* = 7.8 Hz), 7.23-7.64 (m, 10H, arom. H), 7.83 (d, 1H, arom. H, *J* = 8.0 Hz), 7.91 (d, 1H, arom. H, *J* = 7.9 Hz). ^{13}C NMR (600 MHz, $CDCl_3$) *δ* (ppm): 168.07, 160.86, 151.75, 137.26, 136.95, 136.35, 134.98, 134.48, 133.20, 131.93, 130.66, 130.01, 129.47, 127.64, 127.07, 126.01, 125.70, 125.27, 122.97, 122.87, 122.35, 116.89, 46.10, 39.92. Anal. Calcd (%) for $C_{26}H_{15}ClO_3S$: C, 70.51; H, 3.41. Found: C, 70.39; H, 3.54.

(3a*R*,4*S*)-2-Benzyl-4-(3-methoxybenzo[*b*]thiophen-2-yl)-9-phenyl-3a,4-dihydro-1*H*-benzo[*f*]isoindol-1,3(2*H*)-dione (**11**C) and (3a*S*,4*R*)-2-benzyl-4-(3-methoxybenzo[*b*]thiophen-2-yl)-9-phenyl-3a,4-dihydro-1*H*-benzo[*f*]isoindol-1,3(2*H*)-dione (**11**C')

Fulgimide **4***E* (0.04 mmol, 0.02 g) was dissolved at heating in 2.5 mL of CH_3CN in a quartz vessel (5 mL) and irradiated by a Sweko IP65 LED emitter until almost complete decoloration and evaporation. Colorless solid was filtered off, rinsed with methanol (1 mL) and dried. Yield 0.018 g (92%), colorless solid, mp 165-167 ^{o}C. IR ν_{max} (cm^{-1}): 1760, 1706 (C=O). ^{1}H NMR (600 MHz, $CDCl_3$) *δ* (ppm): 4.12 (s, 3H, OMe), 4.18 (d, 1H, CH, *J* = 6.8 Hz), 4.54 (d, 1H, CH_2, *J* = 14.5 Hz), 4.61 (d, 1H, CH_2, *J* = 14.5 Hz), 5.38 (d, 1H, CH, *J* = 6.8 Hz), 6.71-7.65 (m, 17H, arom. H), 7.70 (d, 1H, arom. H, *J* = 7.9 Hz). ^{13}C NMR (600 MHz, $CDCl_3$) *δ* (ppm): 173.73, 166.51, 147.99, 146.86, 139.64, 136.01,

135.54, 134.68, 134.66, 132.26, 130.63, 130.15, 129.38, 129.10, 128.70, 128.02, 127.93, 127.90, 127.72, 127.17, 124.89, 124.67, 124.15, 123.18, 120.89, 120.23, 61.97, 45.39, 41.98, 35.97. Anal. Calcd (%) for $C_{34}H_{25}NO_3S$: C, 77.40; H, 4.78; N, 2.65. Found: C, 77.29; H, 4.59; N, 2.59.

(3a*R*,4*S*)-2-Benzyl-4-(3-chlorobenzo[*b*]thiophen-2-yl)-9-phenyl-3a,4-dihydro-1*H*-benzo[*f*]isoindol-1,3-(2*H*)-dione (**12**C) and (3a*S*,4*R*)-2-benzyl-4-(3-chlorobenzo[*b*]thiophen-2-yl)-9-phenyl-3a,4-dihydro-1*H*-benzo[*f*]isoindol-1,3-(2*H*)-dione (**12**C')

Fulgimide **8***E* (0.04 mmol, 0.02 g) was dissolved at heating in 2.5 mL of CH_3CN in a quartz vessel (5 mL) and irradiated by a Sweko IP65 LED emitter until almost complete decoloration and evaporation. Colorless solid was filtered off, rinsed with methanol (1 mL) and dried. Yield 0.017 g (86%), colorless solid, mp 142-143 ^oC. IR ν_{max} (cm^{-1}): 1761, 1704 (C=O). ^1H NMR (600 MHz, $CDCl_3$) δ (ppm): 4.25 (d, 1H, CH, J = 7.0 Hz), 4.52 (d, 1H, CH_2, J = 14.5 Hz), 4.65 (d, 1H, CH_2, J = 14.5 Hz), 5.55 (d, 1H, CH, J = 7.0 Hz), 6.75-7.09 (m, 3H, arom. H), 7.22-7.55 (m, 13H, arom. H), 7.81 (d, 1H, arom. H, J = 8.4 Hz). ^{13}C NMR (600 MHz, $CDCl_3$) δ (ppm): 173.08, 166.36, 146.81, 138.71, 136.36, 135.75, 135.46, 134.56, 134.49, 133.51, 130.78, 130.37, 129.46, 128.78, 128.56, 128.30, 128.04, 127.92, 127.82, 127.26, 125.66, 125.03, 122.51, 122.17, 119.98, 119.03, 45.19, 42.05, 37.65. Anal. Calcd (%) for $C_{33}H_{22}ClNO_2S$: C, 74.50; H, 4.17; N, 2.63. Found: C, 74.39; H, 4.33; N, 2.59.

References

1S. Zmeeva, S. Yu.; Rybalkin, V. P.; Popova, L. L.; Tkachev, V. V.; Revinskii, Y. V.; Tikhomirova, K. S.; Starikov, A. G.; Dubonosov, A. D.; Bren, V. A.; Aldoshin, S. M.; Minkin, V. I. *Tetrahedron* **2016**, *72*, 5776-5782. doi:10.1016/j.tet.2016.08.002

2S. Ricci A.; Balucani D.; Buu-Hoi N. P. *J. Chem. Soc. C*, **1967**, 779-780. doi: 10.1039/J39670000779

3S. Patel R. M.; Argade N. P. *Synthesis*, **2010**, 1188-1194. doi:10.1055/s-0029-1219233
